# A habitat‐based approach to reporting the direct impacts of an organization on biodiversity

**DOI:** 10.1111/cobi.70071

**Published:** 2025-05-31

**Authors:** Karel Mokany, Chris Ware, Roozbeh Valavi, Katherine Giljohann, Simon Ferrier, Cara Stitzlein, Gonzalo Mata

**Affiliations:** ^1^ CSIRO Canberra Australian Capital Territory Australia; ^2^ CSIRO Sandy Bay Tasmania Australia; ^3^ CSIRO Clayton Victoria Australia; ^4^ CSIRO Floreat Western Australia Australia

**Keywords:** Australia, business, corporate, ecosystem condition, extinction risk, indicator, threatened species, TNFD, Australia, condición del ecosistema, corporativo, especie amenazada, indicador, negocio, riesgo de extinción, TNFD

## Abstract

There is a rapidly growing need for efficient but rigorous methods for organizations to assess and disclose their biodiversity impacts. We devised a habitat‐based analytical approach for estimating the direct impacts of an organization on biodiversity. In our broad approach, we considered the time series of an organization's spatial footprint and assumed its biodiversity position was the accumulated positive and negative impacts over space and time. We demonstrated the approach by assessing the biodiversity position of CSIRO—Australia's national science agency, which has owned or controlled 50 sites across Australia since 1916, covering >460,000 ha. We applied 3 complementary habitat‐based biodiversity indicators (effective habitat area, species extinction risk, and threatened species habitat), all with a fine resolution annual (1987–2023) time series of ecosystem condition as their basis. At the end of the most recent observation year, the CSIRO was in a negative biodiversity position in terms of all 3 biodiversity indicators. Over the time series considered, the activities of CSIRO were estimated to have led to an increase in the extinction risk for all native species by 1.0 species; a reduction in effective habitat area of 11,945 ha and a reduction in threatened species habitat of 22,307 species hectares (i.e., condition‐weighted amount of habitat available to threatened species). Although the magnitude of the biodiversity position for CSIRO was strongly influenced by a single very large site (Murchison), the vast majority of the CSIRO sites were also in a negative position when considered separately. We demonstrated how future‐looking scenario analysis can be linked with this biodiversity assessment approach, with a single natural regeneration action across the large Murchison site estimated to return CSIRO's biodiversity position close to neutral within 50 years.

## Abstract (es)

Un enfoque basado en el hábitat para reportar los impactos directos de una organización sobre la biodiversidad

**Resumen:**

Cada vez es más necesario que las organizaciones dispongan de métodos eficaces y rigurosos para evaluar y divulgar su impacto en la biodiversidad. Diseñamos un enfoque analítico basado en el hábitat para estimar los impactos directos de una organización sobre la biodiversidad. En nuestro enfoque general, consideramos las series temporales de la huella espacial de una organización y asumimos que su posición en materia de biodiversidad son los impactos positivos y negativos acumulados a lo largo del espacio y el tiempo. Demostramos el enfoque evaluando la posición de biodiversidad de CSIRO, la agencia científica nacional de Australia, que posee o controla 50 emplazamientos en todo el país desde 1916, con una superficie de más de 460,000 hectáreas. Aplicamos tres indicadores complementarios de biodiversidad basados en el hábitat (área efectiva de hábitat, riesgo de extinción de especies y hábitat de especies amenazadas), todos ellos con una serie temporal anual de alta resolución (1987‐2023) del estado del ecosistema como base. Al final del año más reciente de observación, la CSIRO se encontraba en una posición de biodiversidad negativa en términos de los tres indicadores de biodiversidad. A lo largo de la serie temporal considerada, estimamos que las actividades de la CSIRO habían provocado un aumento del riesgo de extinción de todas las especies autóctonas en 1.0 especies. También se produjo una reducción de la superficie efectiva de hábitat de 11,945 hectáreas y una reducción del hábitat de especies amenazadas de 22,307 hectáreas de especies (es decir, la cantidad de hábitat ponderada por su estado disponible para las especies amenazadas). Aunque la magnitud de la situación de la biodiversidad en CSIRO estaba muy influida por un solo sitio muy grande (Murchison), la gran mayoría de los sitios de CSIRO también se encontraban en una situación negativa si se consideraban por separado. Demostramos cómo el análisis de escenarios de futuro puede vincularse a este enfoque de evaluación de la biodiversidad, estimando que una sola acción de regeneración natural en el gran sitio de Murchison aproximaría la posición de la biodiversidad de CSIRO a la neutralidad en 50 años.

## INTRODUCTION

There is a growing need for organizations to quantify and disclose their impacts on biodiversity. Historically, organizations focused on responding to regulatory requirements associated with the impact of particular activities on specific aspects of biodiversity, such as the impacts of a development project on threatened species. This approach is quite limited in scope and has done little to protect biodiversity from ongoing decline (Murphy & van Leeuwen, 2021). More recently, broader perspectives, initiatives, and frameworks have emerged that are focused on understanding and disclosing a much wider view of the dependenci and impacts of an organization on biodiversity (Natural Capital Coalition, 2016; SBTN, 2023; TNFD, 2023e). This new focus has been encapsulated in Target 15 of the Kunming–Montreal Global Biodiversity Framework (CBD, 2022): “businesses assess, disclose, and reduce biodiversity‐related risks and negative impacts.”

In this emerging context, organizations around the world have begun committing to being nature positive, to having net positive impacts on biodiversity, or to having no net loss of biodiversity (Addison et al., 2019; zu Ermgassen et al., 2022). Comparison of the biodiversity commitments and performance of organizations is a potentially powerful mechanism that could help bend the curve of ongoing biodiversity loss and decline (Leclère et al., 2020), through sustainability rankings, strategic investment decisions, stakeholder perceptions, and consumer choices.

Organizational biodiversity targets are a useful step forward in moving to reverse the declines in biodiversity globally. However, such targets are unable to be tracked or even clearly articulated without scientific rigor in support. The activities of the Taskforce on Nature‐related Financial Disclosures (TNFD) have been beneficial in establishing broad guidance on methods and metrics for quantifying the biodiversity impacts of an organization (TNFD, 2023c). However, frameworks such as this do not prescribe specific assessment methods and data sources, given the diverse capabilities and circumstances of different organizations operating in different parts of the world. The TNFD also recognizes that a single indicator or metric will generally be insufficient to monitor and report impacts on biodiversity and nature, given the various ways in which biodiversity can be considered and quantified (Magurran & McGill, 2010).

To date, a relatively small number of approaches have been published in the scientific literature for the specific needs of organizational biodiversity assessment (Bull et al., 2022), though a variety of tools and techniques are emerging (Lammerant et al., 2022; TNFD, 2023a; Zhu et al., 2024). These approaches have generally applied remote assessments to infer likely biodiversity impacts based on spatial data on changes in habitat for biodiversity, though typically not at fine spatial and temporal scales. On‐ground observations of biodiversity change, such as species population sizes, are often considered the highest quality information, but for many organizations, such data will be difficult to obtain and interpret for corporate reporting.

**FIGURE 1:**
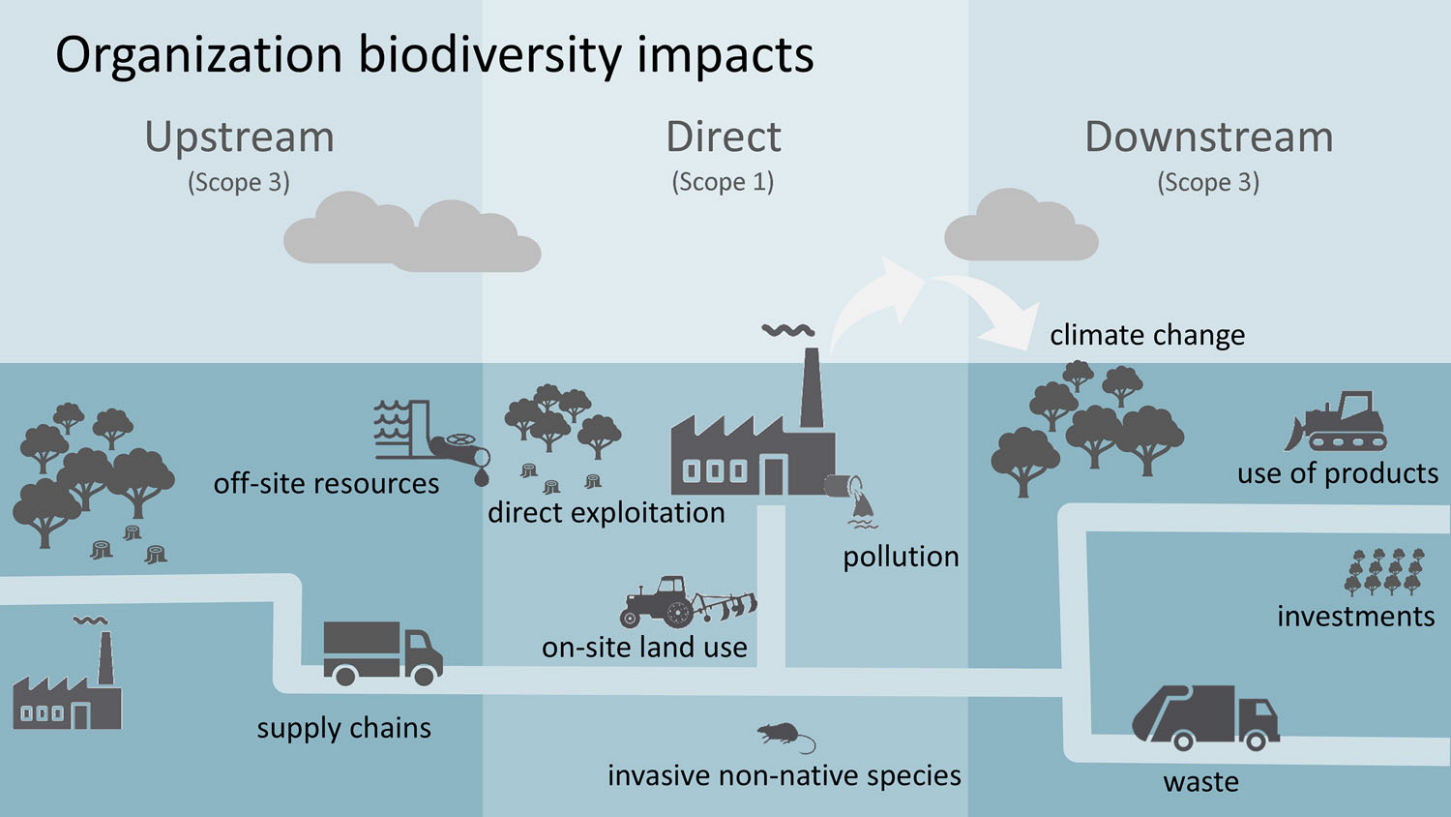
The range of impacts an organization may have on biodiversity. The scope of biodiversity impacts is adapted from the levels applied in reporting greenhouse gas emissions (WRI & WBCSD, 2004).

There are a variety of ways in which an organization may impact biodiversity (Figure 1). These include direct impacts (Scope 1) associated with the organization's operations on areas they own or control and indirect impacts (Scope 3) associated with upstream resource inputs (supply chains) and downstream product use, investments, and waste (Figure 1). We focused on the direct impacts of an organization in the areas they control, own, or operate and considered impacts on biodiversity that result from the organization influencing ecosystem condition (e.g., habitat loss, degradation or restoration).

**FIGURE 2:**
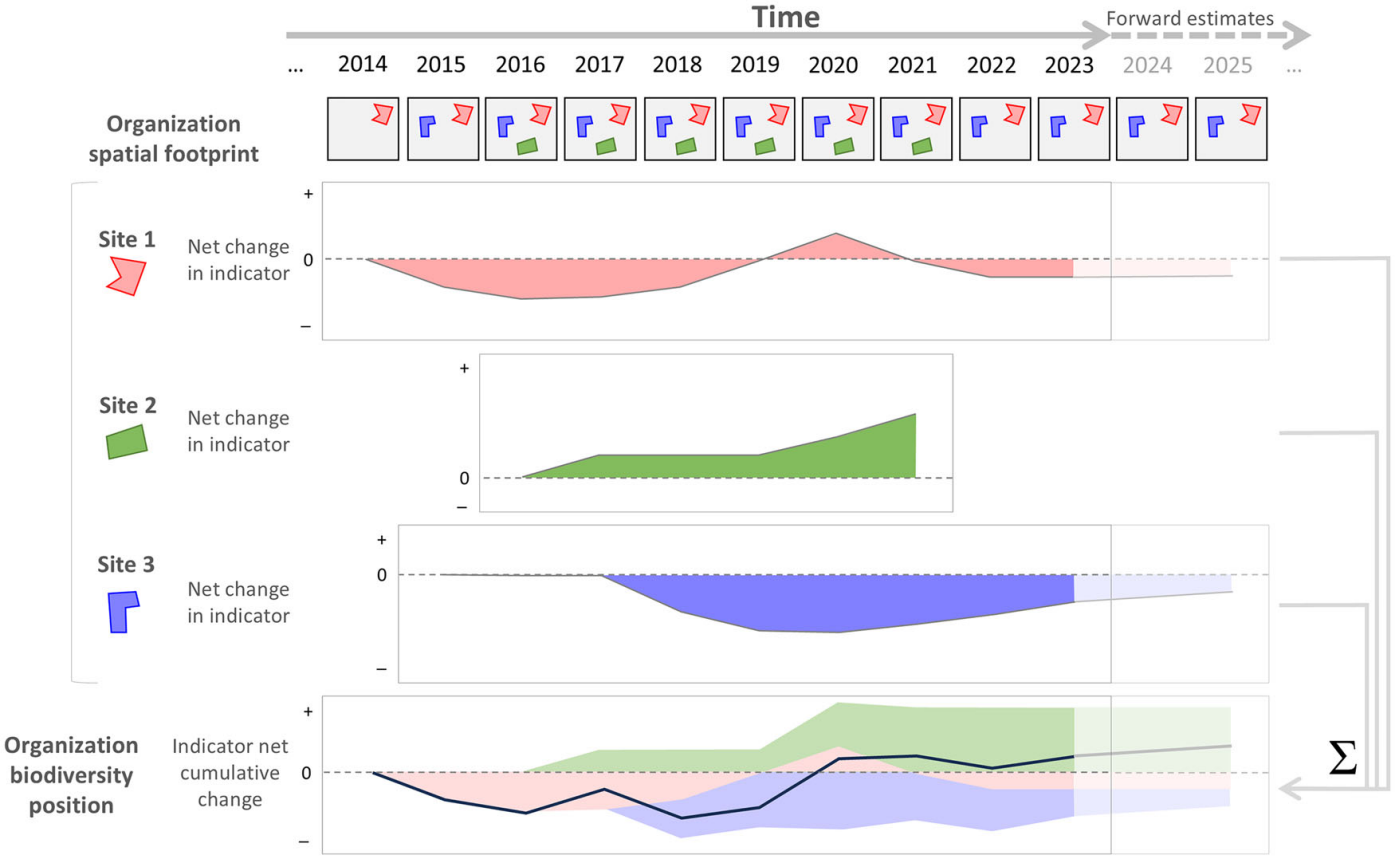
An approach to quantifying an organization's biodiversity position for biodiversity indicators that are additive across sites (polygons), such as effective habitat area (i.e., condition‐weighted area), that can be derived at an organization level (black line) by summing the impacts of individual sites. For the whole organization, the net cumulative change in the indicator represents its biodiversity position, being the summed organizational net change from all sites in prior years. Sites that the organization relinquishes control over (i.e., Site 2 above) continue to contribute to the organization's biodiversity position based on the net change in the indicator value (relative to baseline) in the final year of site ownership.

**FIGURE 3:**
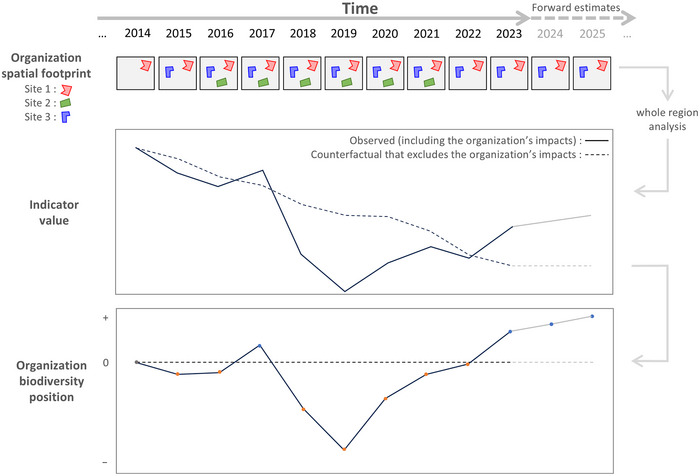
An approach to quantifying an organization's biodiversity position for biodiversity indicators that are nonadditive across sites, such as species extinction risk or ecosystem connectivity, and that cannot be derived at an organization level by summing the impacts of individual sites. Quantifying the biodiversity position of the organization requires a whole‐region analysis, where the observed change in the indicator is contrasted with a counterfactual scenario that removes all impacts of the organization over time on ecosystem condition. The biodiversity position of the organization is positive when the observed indicator value for the whole region is greater than the counterfactual scenario excluding the organization's impacts (the world without the organization).

We devised and applied an analytical approach for habitat‐based biodiversity indicators that considers the time series of an organization's spatial footprint and derives the biodiversity position for an organization as the accumulated positive and negative impacts. This represents a novel and powerful approach to organizational biodiversity assessment, contrasting with the simpler approach that considers change in a biodiversity metric for each year independently. We applied this approach for additive biodiversity indicators, such as ecosystem condition (Figure 2), and nonadditive indicators, such as species extinction risk, that depend on attributes of the surrounding region (Figure 3). We demonstrated this approach by quantifying the biodiversity position of CSIRO (the Commonwealth Scientific and Industrial Research Organization, Australia) and demonstrated how it can be used in an integrated way to link monitoring of biodiversity position with forecasting of likely future positions under proposed ecosystem management actions.

## METHODS

### Broad approach to organizational biodiversity assessment

Our approach to assessing an organization's biodiversity position considers the history of the spatiotemporal footprint of areas (polygons) the organization has owned or controlled (Figures 2 & 3). The temporal span of each polygon in the organization's spatial footprint is important, given that, for example, large negative impacts on biodiversity may occur early in the ownership or control of an area, with very little impact in subsequent years (given there may be no more biodiversity left to impact). For the habitat‐based biodiversity assessment approach implemented here, the organizational footprint must be specified as spatial polygons that do not overlap in both space and time. Spatial overlap of footprint polygons is permitted only where they do not overlap the period occupied by the organization. Ensuring that there are no spatiotemporal overlaps in the organizational footprint avoids double counting increases or decreases in the biodiversity indicators.

For each polygon, the time point prior to when the organization assumed control is used as the baseline against which subsequent change is considered (i.e., the year prior, for annual data). Where the organization assumed control of a polygon prior to the commencement of the data time series, the first year of available data is used as the baseline, thereby explicitly ignoring any prior positive or negative impacts.

In each polygon, changes to the biodiversity indicators are considered up until the year the organization relinquishes control of that polygon. Any further changes to ecosystem condition that occur after the organization has relinquished control/ownership of a polygon do not contribute to changes in the organization's biodiversity position. However, the total impacts (positive or negative) accrued by the organization in a polygon over the time it was controlled/owned continue to contribute thereafter to the organization's biodiversity position by that constant amount. Hence, past negative (or positive) biodiversity impacts in a polygon are not omitted from the organization's biodiversity position when that polygon is sold.

The way in which the organizational biodiversity assessment considers the combination of spatial footprint polygons over time differs, depending on whether the biodiversity indicator is additive or nonadditive. Additive biodiversity indicators, such as ecosystem condition, are where the value for each location (grid cell) is based only on data for that grid cell, and summary values across one or more polygons can be obtained simply by summing the grid cell values (Figure 2). Nonadditive biodiversity indicators, such as species extinction risk or habitat connectivity, are those for which quantifying the impact of an organization needs to consider not only the polygons owned or controlled by the organization but also other areas across the surrounding region. For nonadditive biodiversity indicators, a regional analysis is required rather than simple summary (addition) across the grid cells in the footprint polygons. That regional analysis compares the biodiversity indicator value resulting from the observed time series, with the biodiversity indicator value resulting from a counterfactual time series that assumes the organization had no impact on ecosystem condition (Figure 3).

We prepared data for and applied 3 biodiversity indicators: effective habitat area, a spatial summary of ecosystem condition; species extinction risk, the number of native species at risk of extinction; and threatened species habitat, the condition‐weighted amount of habitat available to threatened species. Each indicator was based on the assumption that the estimated ecosystem condition in a location (grid cell) is indicative of the quality of habitat available for biodiversity.

### Organizational footprint data preparation

Specifying the spatial and temporal footprint of the organization is the first important data requirement in assessing the impacts of the organization on biodiversity. This is equivalent to the locate phase of the TNFD's LEAP approach (TNFD, 2023d). As an organizational case study, we focused on CSIRO—Australia's national science agency, which commenced operations in 1916 and has operational sites in every Australian state and territory (Appendix S1). The CSIRO was selected as the case study given its long history and diverse range of sites. Land use across CSIRO sites ranges from office buildings surrounded by natural, seminatural, or highly modified environments to agricultural research stations used for intensive cropping and livestock grazing and larger properties used to house radioastronomy infrastructure with grazing on surrounding areas. Spatial boundaries (polygons) were obtained for every currently owned or controlled site and as many of the historic sites no longer owned or controlled as possible. Locations where CSIRO leases part of a building were excluded from the analyses. For each polygon, we used available records to identify the year in which CSIRO took ownership or control and, where relevant, the year in which CSIRO relinquished ownership or control (Appendix S2).

### Ecosystem condition indicator data preparation

Ecosystem condition is the capacity of an area to provide the structure and functions necessary for the persistence of all species naturally expected to occur in that area if it were in an intact (reference) state (Williams et al., 2021). Ecosystem condition values vary continuously from 0 to 1 (or 0–100%). A value of 1 (100%) indicates the ecosystem is in an intact reference state (i.e., it has high levels of ecological integrity), and 0 means there is no capacity for naturally occurring species to persist. The estimated ecosystem condition data applied here are an annual time series (1987–2023) at approximately 100‐m resolution (0.001 degrees), covering continental Australia and nearby islands (Appendix S3).

### Ecosystem condition indicator organizational analyses

The estimated ecosystem condition (*c_i_*_,_ *_t_*) across all *n* grid cells in a polygon (*k*) in any year *t* is summarized as the effective habitat area (*H_k_*_,_ *_t_*) (Ferrier & Drielsma, 2010). This is the effective area of habitat (in hectares) being provided to biodiversity, accounting for continuous variation in ecosystem condition between grid cells, and is calculated as

(1) $$H_{k,t}=\sum_{i=1}^{n}c_{i,t}a_{i},$$

where *c_i_*_,_ *_t_* is the ecosystem condition value of grid cell *i* (expressed in a 0–1 range) in year *t* and *a_i_* is the area in hectares of grid cell *i* that is covered by polygon *k*. A range of alternate names for effective habitat area have been used, including “habitat hectares” (Parkes et al., 2003) and “weighted area” (Bull et al., 2014).

The net change in effective habitat area ($\Delta H_{k,t_{w}}$) for polygon *k* in year *w* (*t_w_*) is taken as the difference from the baseline year (*t*_0_), which is the year prior to the organization taking control of the polygon:

(2) $$\Delta H_{k,t_{w}}=H_{k,t_{w}}-H_{k,t_{0}}.$$

where an organization has relinquished control of polygon *k* prior to *t_w_*, the final year of control is used in place of *t_w_*. The net change in effective habitat area for polygon *k* ($\Delta H_{k,t_{w}}$) is assumed to represent the impact of the organization on ecosystem condition in that polygon. To determine the impact of all *q* polygons across the entire organizational footprint on ecosystem condition in year *w* ($\Delta H_{t_{w}}$ [net cumulative change in effective habitat area in hectares]), the net change values for each polygon *k* are simply summed:

(3) $$\Delta H_{t_{w}}=\sum_{k=1}^{q}\Delta H_{k,t_{w}}.$$

### Species extinction risk indicator data preparation

Spatial layers of estimated community‐level biodiversity patterns underpin the species extinction risk indicator. The analytical method is described by Ferrier et al. (2004) and has been applied in a range of studies (Allnutt et al., 2008; Di Marco et al., 2019; Mokany, Ware, et al., 2022; UNEP‐WCMC, 2016). The objective of this analysis was to estimate the proportion of originally occurring species expected to persist indefinitely into the future given the ecosystem condition across the entire analysis region.

There are 2 key input data sets for the community‐level approach to estimating species extinction risk. The first inputs are spatial layers from a generalized dissimilarity model (GDM) (Ferrier et al., 2007). These are used to estimate the expected level of species assemblage similarity (community compositional similarity) between any pair of locations if those locations were still in reference condition (i.e., highest possible level of ecological integrity). We applied a GDM for vascular plants across Australia that was derived using data from 118,509 plant community survey plots and projected at ≈100‐m resolution (deviance explained 32.4% with 8 environmental predictors and geographic distance) (Mokany, McCarthy, et al., 2022a, 2022b). The second input data are a spatial layer of expected species richness (number of species in an area) under reference ecosystem condition. We used a species richness model for vascular plants across Australia that was derived using the same data as the GDM described above and projected at ≈100‐m resolution (deviance explained 33.4% with 9 environmental predictors) (Mokany, McCarthy, et al., 2022a, 2022b).

### Species extinction risk indicator organizational analyses

For any given spatial layer of ecosystem condition, the spatial layers of estimated community‐level biodiversity patterns (community compositional similarity and species richness) under reference conditions are used to estimate the total proportion of species likely to persist over the long term over a region (*P*) if the ecosystem condition patterns were to remained constant (Ferrier et al., 2004; Mokany et al., 2019). Specifically, for each and every grid cell *i*, we estimated the proportion (*p_i_*) of species historically occurring in this cell (pre‐intensification) that are likely to persist in the remaining habitat anywhere in their range (Allnutt et al., 2008):

(4) $$p_{i}={\left[\sum_{j=1}^{n}s_{ij}c_{j}/\sum_{j=1}^{n}s_{ij}\right]}^{z},$$

where *s_ij_* is the predicted compositional similarity between the focal grid cell *i* and each grid cell *j* in the region of *n* grid cells (from the model of compositional dissimilarity), *c_j_* is the ecosystem condition in each grid cell *j* (ranging continuously from intact reference condition [1] to fully degraded habitat [0]), and *z* is the exponent of the species–area relationship. For grid cell *i*, Σ*s_ij_* quantifies the amount of similar habitat across the region if all grid cells were in reference condition (i.e., the highest possible level of ecological integrity), whereas Σ*s_ij_c_j_* quantifies the amount of similar habitat across the region, accounting for habitat loss and degradation in some grid cells (through *c_j_*). The proportion of remaining similar habitat is converted to an estimate of the proportion of species persisting (*p_i_*) by invoking the species–area relationship, for which we applied a *z*‐value of 0.25, which approximated values commonly observed for terrestrial taxa (Rosenzweig, 1995).

The overall proportion of species expected to persist across a region (*P*) were then estimated as a weighted average of the *p_i_* values for all *n* individual grid cells to incorporate the effects of compositional overlap between grid cells and the species richness of each cell (*m_i_*):

(5) $$P=\sum_{i=1}^{n}p_{i}v_{i}/\sum_{i=1}^{n}v_{i},$$

where the weight (*v_i_*) of a grid cell is calculated as

(6) $$v_{i}=\frac{m_{i}}{\sum_{j=1}^{n}s_{ij}}.$$

This approach is applied to estimate the proportion of the original species across the analysis region that are expected to persist, given spatial patterns in ecosystem condition. The expected proportion of originally occurring native species across the region at risk of extinction (E_p_) is derived as the complement of the expected proportion of species persisting (i.e., E_p_ = 1 − *P*). This proportion of originally occurring species at risk of extinction can then be converted into units of number of species at risk of extinction (E_n_) by multiplying by the estimated number of originally occurring species in the analysis region (γ) (i.e., E_n_ = E_p_γ). For the present assessments, we considered the entire continent of Australia, to which we applied an estimated 500,000 originally occurring (pre‐European) native species (Chapman, 2009).

To determine the impact of an organization on the number of species at risk of extinction, the estimated species extinction risk for the observed historic time series of ecosystem condition is compared to an estimate based on a counterfactual scenario of ecosystem condition that removes the impacts of the organization. Under this counterfactual scenario, there are no changes in ecosystem condition in a footprint polygon once an organization takes control of that polygon. That is, the ecosystem condition value for a grid cell *i* in year *w* ($c_{i,t_{w}}$) is set at the observed value in the baseline year (*t*_0_), which is the year prior to the organization taking control of the polygon. After the organization relinquishes control of a polygon, the ecosystem condition value for a grid cell in that polygon is set at the baseline value plus any change in condition following the year the organization relinquishes control of that polygon (*t_e_*). Specifically, for any grid cell *i* in any footprint polygon *k*, the counterfactual ecosystem condition value in year *t_w_* ($c_{i,t_{w},\mathrm{count}}$) is determined by

(7) $$c_{i,t_{w},\mathrm{count}}=\left\{\begin{array}{c} c_{i,t_{w}},\\ c_{i,t_{0}},\\ c_{i,t_{0}}+\left(c_{i,t_{w}}-c_{i,t_{e}}\right), \end{array}\right.\;\;\;\;\;\begin{array}{c} \mathrm{if}\;t_{w}\leq t_{0},\\ \mathrm{if}\;t_{0}<t_{w}\leq t_{e},\\ \mathrm{if}\;t_{w}>t_{e}. \end{array}$$

For all grid cells *i* outside the organization footprint, the counterfactual ecosystem condition in year *t_w_* is simply the observed value of ecosystem condition for that grid cell in that year (i.e., $c_{i,t_{w},\mathrm{count}}$ = $c_{i,t_{w}}$). This process for generating a counterfactual time series of ecosystem condition essentially removes the impact of the organization on any changes observed in ecosystem condition, so it represents an estimate of the full analysis region without the organization's impacts. The additive indicators (ecosystem condition, threatened species habitat) effectively apply a similar counterfactual approach, but given that only grid cells in the footprint polygons are considered for those indicators, it is simpler to express their calculation as a comparison to baseline year. Given that calculation of species extinction risk also considers ecosystem condition outside the footprint polygons, the counterfactual puts a baseline (in the polygons) in the context of ongoing change outside the footprint polygons.

Finally, to obtain the estimated impact of an organization on species extinction risk (*E_n_*_,org_), we take the difference between the estimated species extinction risk based on the observed ecosystem condition layer (*E_n_*_,obs_) and the estimated species extinction risk based on the counterfactual ecosystem condition layer (*E_n_*_,count_):

(8) $$E_{n,\mathrm{org}}=E_{n,\mathrm{obs}}-E_{n,\mathrm{count}},$$

where positive values indicate more species at risk of extinction due to the impacts of the organization on ecosystem condition, negative values indicate a reduction in species extinction risk, and a value of zero indicates no effect of the organization on species extinction risk. This indicator is calculated each year, noting that the derivation of the counterfactual ecosystem condition time series of spatial layers incorporates impacts of an organization on ecosystem condition since they took ownership or control of each polygon. Given that the key indicator for organizational reporting is the net change in species extinction risk (*E_n_*_,org_), if an organization makes no change to ecosystem condition within their footprint, the value of this indicator will be zero, even though the changes in ecosystem condition outside the footprint are considered in its calculation.

### Threatened species habitat indicator data preparation

To derive an indicator of habitat provision for threatened species, we applied a data set at ≈100‐m resolution across Australia on the estimated spatial distribution of Australia's nationally listed terrestrial threatened species (excluding migratory species) (Giljohann et al., 2022). In summary, spatial data on species’ estimated current extent of occurrence were refined from the likely‐to‐occur spatial distribution of each species, published by the Australian Department of Climate Change, Energy, the Environment and Water (DCCEEW, 2022). For each species (*n* = 1518), areas in the likely‐to‐occur distribution (DCCEEW, 2022) were restricted using filtered occurrence observations from the Atlas of Living Australia (Atlas of Living Australia, 2022). We retained only those grid cells that were within the observed elevational limits (Geoscience Australia, 2008) of the occurrence data and grid cells where the combined IBRA bioregion (DCCEEW, 2020) and NVIS pre‐European major vegetation type (DCCEEW, 2021) were represented in the occurrence observations.

### Threatened species habitat indicator organizational analysis

We estimated the capacity of habitat to support threatened species (*T_i_*_,_ *_t_*) in each grid cell *i* in year *t* by multiplying the ecosystem condition of that grid cell (*c_i_*_,_ *_t_*) in year *t* (expressed in a 0–1 range) by the number of threatened species (*q_i_*) for which that grid cell could potentially form part of their habitat:

(9) $$T_{i,t}=\left\{\begin{array}{c} c_{i,t}q_{i},\\ 0, \end{array}\;\;\;\begin{array}{c} \mathrm{if}\;c_{i,t}\geq 0.5,\\ \mathrm{otherwise}, \end{array}\right.$$

where *q_i_* is derived by combining the estimated potential distribution of all threatened species. Here, we assumed that any location with low ecosystem condition (<0.5) provides no habitat for threatened species and that above this value the benefit of the habitat increases with the ecosystem condition value. Previous assessments have been relatively insensitive to this arbitrary threshold (Giljohann et al., 2025), though further research is required to better understand how threatened species populations vary in locations with different ecosystem condition. Although species will vary in the degree to which areas of different ecosystem condition support their populations, we assumed that this is the average response.

The estimated capacity of habitat to support threatened species across all *n* grid cells *i* in a polygon (*k*) in any year *t* is calculated as

(10) $$T_{k,t}=\sum_{i=1}^{n}T_{i,t}a_{i},$$

where *a_i_* is the area (ha) of grid cell *i* that is covered by polygon *k*. Capacity of habitat to support threatened species habitat provision in a polygon is quantified in units of species hectares (species·ha), which integrates the amount and quality of habitat and how many threated species could potentially occur. For example, a single hectare of habitat in intact reference condition (1.0 or 100%) where 10 threatened species potentially occur would provide 10 species·ha, and a 20‐ha area in moderate ecosystem condition (0.5 or 50%) covering the potential distribution of a single threated species would also provide 10 species·ha of threated species habitat.

Given the additive nature of this biodiversity metric, we used the same approach as with ecosystem condition to determine the impact of the organization on threatened species habitat area in year *w* (*t_w_*) across all *q* polygons in their footprint. Specifically, we first determined the net change in threatened species habitat ($\Delta T_{k,t_{w}}$) for that polygon from the baseline year (*t*_0_), being the year prior to the organization taking control of the polygon, as

(11) $$\Delta T_{k,t_{w}}=T_{k,t_{w}}-T_{k,t_{0}}.$$

Where an organization relinquished control of polygon *k* prior to *t_w_*, the final year of control (*t_e_*) is used in place of *t_w_*. The net change in threatened species habitat for polygon *k* ($\Delta T_{k,t_{w}}$) represents the impact of the organization on threatened species habitat in that polygon. To determine the impact of the entire organizational footprint on threatened species habitat in year *w* ($\Delta T_{t_{w}}$ [net cumulative change in threatened species habitat, in species·ha]), the net change values for each polygon *k* for year *t_w_* are summed:

(12) $$\Delta T_{t_{w}}=\sum_{k=1}^{q}\Delta T_{k,t_{w}}.$$

This approach to estimating threatened species habitat does not upweight the contribution of threatened species that have a very small area of potential habitat; hence, threatened species with larger areas of potential habitat will more frequently contribute to nonzero values of estimated threatened species habitat. Threatened species with smaller areas of potential habitat are upweighted in alternative threatened species habitat metrics, such as the Species Threat Abatement and Restoration Metric (STAR) (Mair et al., 2021). We did not apply the STAR metric because it is primarily a prioritization indicator rather than a change monitoring indicator.

### Estimating changes into the future

Considering how the biodiversity position of the organization is likely to change in the future is important in enabling decisions that lead to improved outcomes for biodiversity and for an organization to plan actions that will lead to the achievement of established goals and targets (TNFD, 2023b). Estimating change into the future can consider the likely effects of actions that have already been implemented, such as restoration actions, where the benefits for biodiversity are likely to continue to accrue over time as the restored ecological community matures. This likely future position under already implemented actions can be contrasted with alternative futures where one or more proposed actions are considered.

When using a habitat‐based approach to biodiversity assessment, future estimates require the translation of one or more actions in a specified area at a specified time into a future scenario of ecosystem condition. This enables reporting of the estimated future biodiversity position of the organization for each habitat‐based biodiversity indicator. Our analytical approach to estimating future ecosystem condition under alternative management actions is described in Appendix S4 and involved implementing a response function for each action based on the most recent ecosystem condition observation and parameters inferred by best available evidence. In assessing the potential implications of proposed or implemented ecosystem management actions on an organization's biodiversity position, the same analytical methods for indicator quantification were applied as described above, but we used the forecast ecosystem condition spatial layers in place of the observed ecosystem condition spatial layers.

## RESULTS

Information collated on the history of CSIRO's spatiotemporal footprint includes 50 sites across Australia and covers the period from 1924 to 2023. Sites ranged in size from 0.14 ha (Notting Hill) to 451,704 ha (Murchison) (Appendices S1 & S2). The Murchison site by itself accounts for 98% of the land area of all CSIRO's historic and current sites.

**FIGURE 4:**
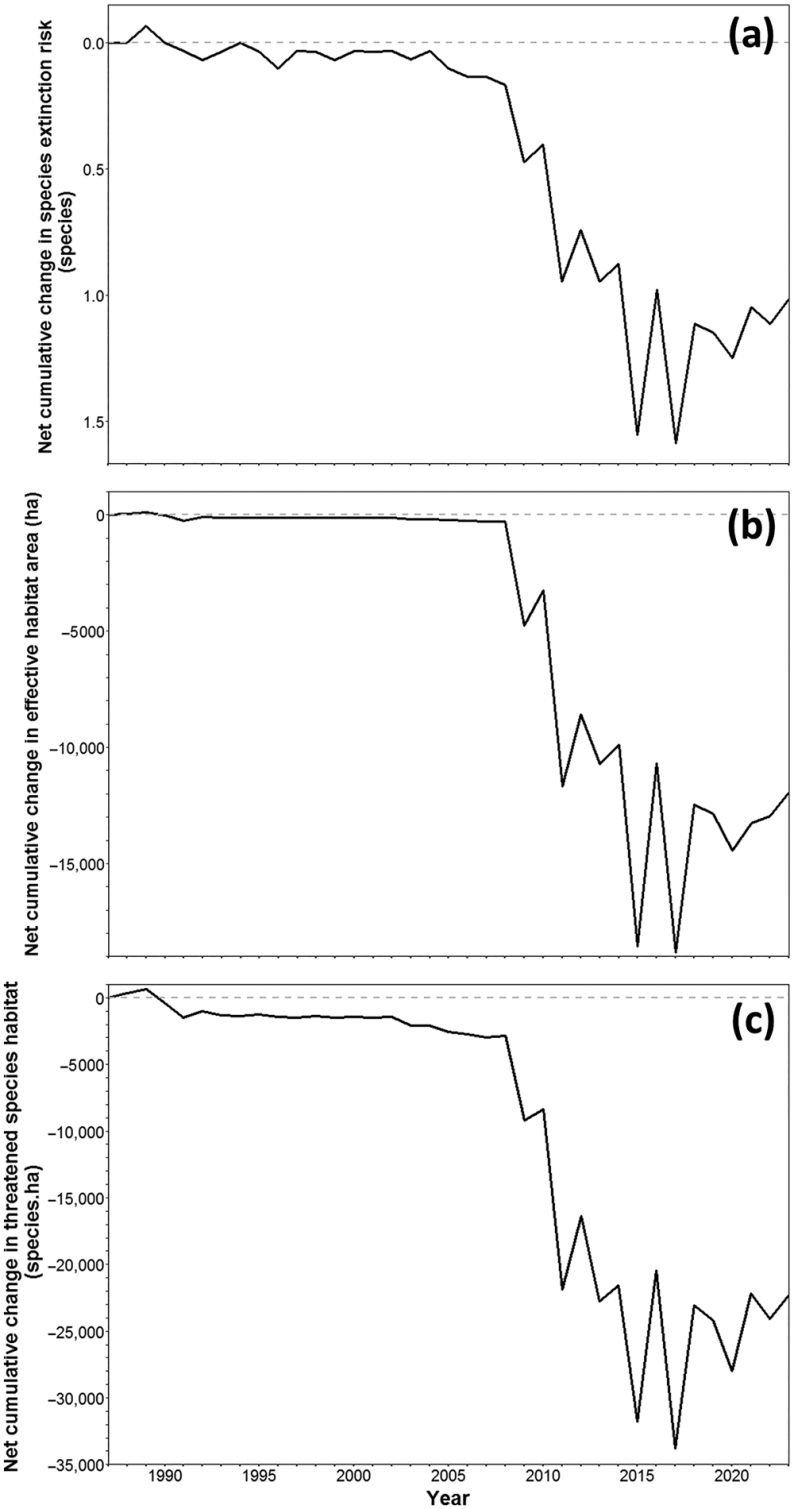
Estimated change in CSIRO's biodiversity position from 1987 to 2023 for 3 indicators: (a) species extinction risk, (b) effective habitat area, and (c) threatened species habitat (values above 0 indicate a position of no net loss of biodiversity; values below 0 indicate the organization has had negative impacts on biodiversity). This assessment ignores positive or negative impacts prior to the commencement of collection of remotely sensed ecosystem condition data in 1987.

**FIGURE 5:**
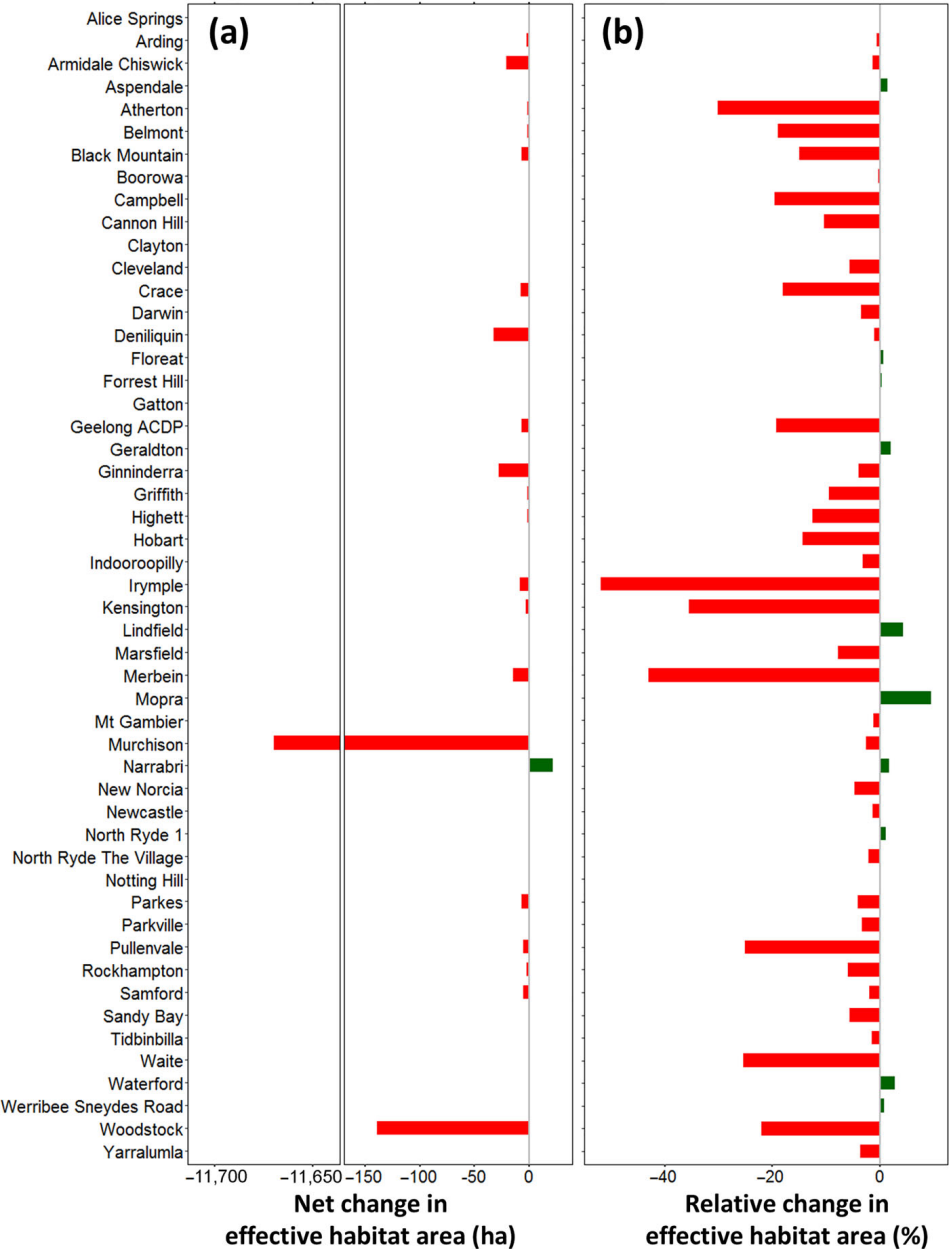
(a) Contribution of each CSIRO site to the biodiversity position of CSIRO in terms of the effective habitat area indicator and (b) net change in effective habitat area relative to the area of each CSIRO site (negative values, sites had negative impacts on biodiversity).

With respect to all 3 biodiversity indicators, our assessment indicated that CSIRO was in a negative biodiversity position in terms of direct impacts as of 1 January 2024 (Table 1; Figure 4). This most recent estimate indicated that past activities on CSIRO sites increased the number of Australian native species at risk of extinction by 1.0 species; decreased the effective area of habitat available to biodiversity by 11,945 ha through reductions in ecosystem condition; and reduced the amount of threatened species habitat by 22,307 species·ha (Table 1; Figure 4). Although CSIRO's biodiversity position was predominantly influenced by the very large Murchison site, 34 of the 50 sites had a negative biodiversity position in terms of the ecosystem condition indicator (effective habitat area) as of 1 January 2024 (Figure 5; Appendix S2). When assessed relative to the area of each site, Murchison had a low relative change in effective habitat area (−2.6%) compared to much smaller sites such as Irymple (−51.9% over 15 ha) and Merbein (−43.0% over 34 ha) (Figure 5). Changes in biodiversity position over time were also compared across sites (Appendix S5) and highlighted the timing of the largest changes in site‐level biodiversity position.

**TABLE 1:** Net cumulative change for 3 biodiversity indicators^a^ used to estimate change in the biodiversity position for CSIRO from 2022 to 2023.

Year	Net cumulative change in species extinction risk (species)	Net cumulative change in effective habitat area (ha)	Net cumulative change in threatened species habitat (species·ha)^b^
2022	+1.11	−12,968	−24,045
Change^c^	−0.10	+1023	+1738
2023	+1.01	−11,945	−22,307

^a^ A negative biodiversity position within a given year is indicated by negative values for the net cumulative change in effective habitat area and threatened species habitat and positive values in the net cumulative change in species extinction risk.

^b^ Threatened species habitat combines the amount of condition‐weighted habitat with the number of threatened species supported by it.

^c^ Change in the biodiversity position between years.

**FIGURE 6:**
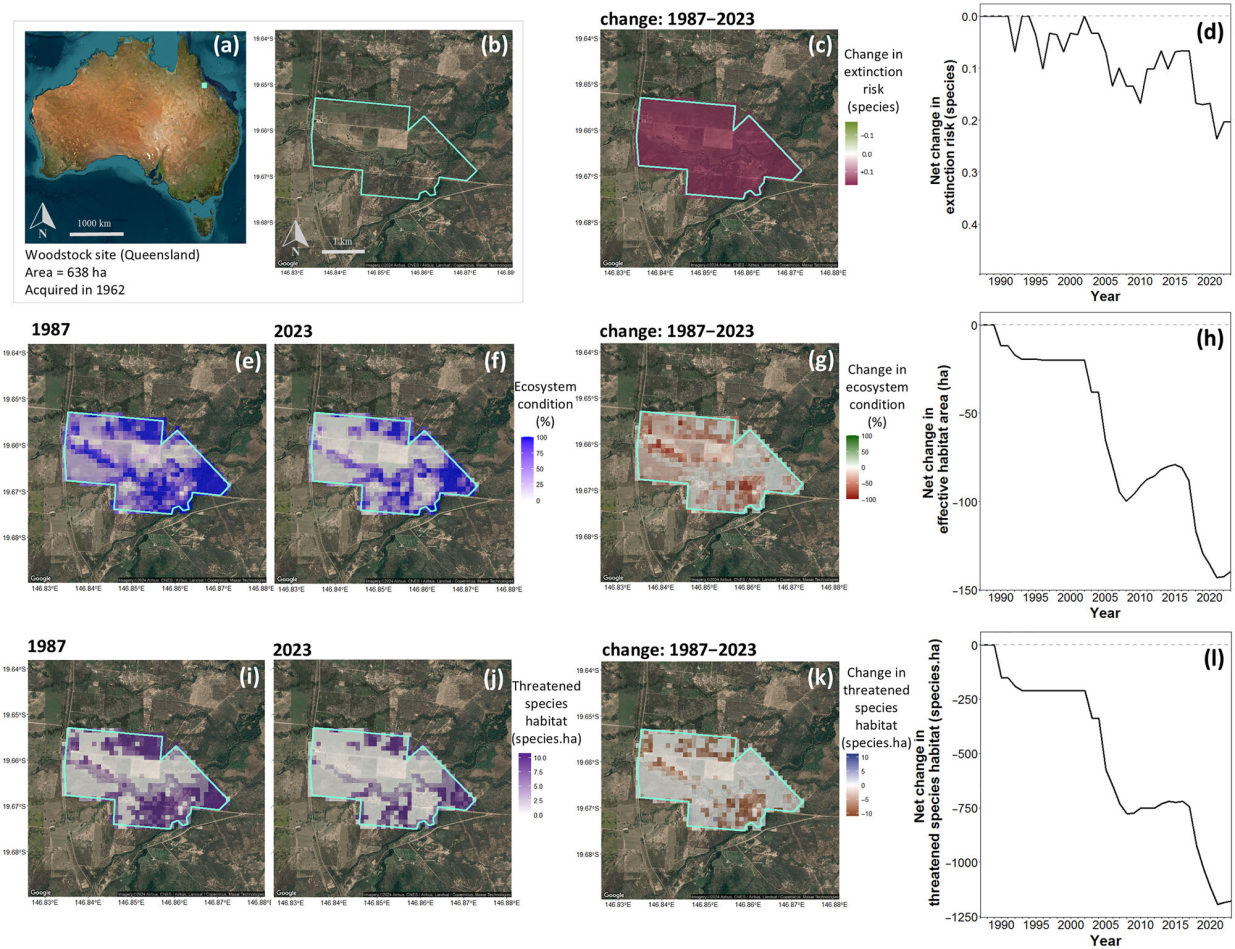
Site‐level biodiversity assessment outcomes for CSIRO's Woodstock site in Queensland, Australia, including (a, b) contextual information, (c, d) species extinction risk, (e–h) ecosystem condition (and effective habitat area), and (i–l) threatened species habitat. Maps show raster data for the site for the (e, i) earliest available year (1987), the (f, j) most recent available year (2023), and the (c, g, k) change over the time series. Change in the site‐level biodiversity position for the 3 indicators over time is also shown (d, h, l), where values below 0 indicate that the site had negative impacts on biodiversity. Background imagery is map data from Google 2024.

The finely resolved spatial data underlying the organizational biodiversity assessment enabled interrogation of the biodiversity position of individual sites, as demonstrated for CSIRO's Woodstock site, used for agricultural research (Figure 6). The 3 site‐level biodiversity indicators all estimated a negative biodiversity position for the Woodstock site (Figure 6d,h,l). The spatial data on which the indicators were based identified the greatest reductions in ecosystem condition and threatened species habitat in the northwest and southeast areas of the Woodstock site (Figure 6g,k).

**FIGURE 7:**
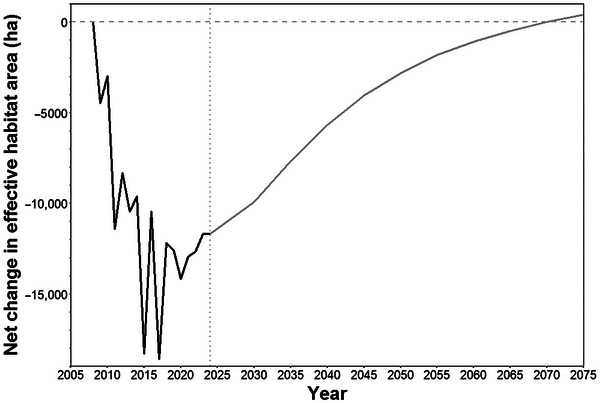
Estimated net change in the effective habitat area indicator for CSIRO's Murchison site from the year of acquisition (2009) to the most recent observation year (2023) (dark black line) and the expected change up to 2075 under the scenario of natural regeneration management implemented across the entire site from 2024 (gray line) (values above 0, position of no net loss of biodiversity; values below 0, site had negative impacts on biodiversity).

The capacity to combine past‐to‐present monitoring data with present‐to‐future scenario analysis was demonstrated for the large Murchison site, given its importance in influencing the organizational biodiversity position for CSIRO. We assessed a single scenario, implementing a natural regeneration action across the entire site, equivalent to removing all livestock and controlling any feral herbivores to allow the existing native vegetation to regenerate naturally (Appendix S4). We estimated that this action would by 2075 (≈50 years) lead to a reduction in species extinction risk by 0.85 species, to an improvement in ecosystem condition leading to an additional 12,062  ha of effective habitat area (Figure 7), and to an increase of 19,212 ha of threatened species habitat. This one action therefore has the potential to move CSIRO much closer to a net neutral biodiversity position compared to the latest estimate (Table 1).

## DISCUSSION

### Organizational biodiversity assessment

Scientifically rigorous organizational biodiversity assessment methods are critical to meeting the growing needs for organizations to reliably quantify and disclose their impacts on biodiversity. Although there are an increasing array of methods and tools emerging for corporate biodiversity assessment (Lammerant et al., 2022; TNFD, 2023a; Zhu et al., 2024), there is a lack of peer‐reviewed scientific publications presenting clear, repeatable, and scalable methods and case studies (e.g., Bull et al., 2022). Reliable and timely information on an organization's biodiversity impacts is crucial for meaningful progress on halting and reversing declines in biodiversity.

We have presented a generic biodiversity assessment method that considers the history of direct impacts of an organization on biodiversity (Figures 2 & 3). This cumulative view is important, given that the vast majority of the direct impacts of an organization on biodiversity could occur in a single year, for example, if all habitat was cleared from the sites owned. Our organizational assessment approach treats biodiversity in a similar way to financial gains and losses: they accumulate over time resulting in either ongoing debt or credit (Houdet et al., 2020).

We implemented the organizational biodiversity assessment approach by using 3 biodiversity indicators, derived from fine‐resolution spatial data covering Australia. In this habitat‐based assessment approach, ecosystem condition, summarized through the effective habitat area indicator, provided the most basic information on biodiversity impacts of an organization. The other 2 indicators we applied combine the ecosystem condition data with information on spatial biodiversity patterns, which is important because the same change in ecosystem condition can lead to very different levels of biodiversity impact, depending on where those changes occur. All 3 biodiversity indicators accounted for the change due to the impact of an organization's activities on ecosystem condition. These indicators do not rely on direct observation of changes in species occurrence or abundance, which are often influenced by factors outside the control of the organization.

Including nonadditive biodiversity indicators when assessing the impacts of an organization is also important to account for the context within which the organization is operating. We estimated the impact on the extinction risk of all native species, based on the Biodiversity Habitat Index method, which is a component indicator under the Kunming–Montreal Global Biodiversity Framework (CBD, 2022). This indicator accounts for patterns in species composition and ecosystem condition outside the spatial boundaries of an organization, thereby quantifying the contribution of the organization to the cumulative impact on extinction risk (Mokany et al., 2019). The TNFD has identified nonadditive indicators, such as extinction risk and connectivity, as important disclosure metrics for organizations (TNFD, 2023c, 2023d). Considering nonadditive habitat‐based indicators in an organizational biodiversity assessment simply requires an approach to prepare a counterfactual spatiotemporal scenario of ecosystem condition that excludes the impacts of the organization, against which to compare the observed changes (see “METHODS”).

### Demonstration biodiversity assessment for CSIRO

Our demonstration assessment for CSIRO showed that the organization was estimated to be in a negative biodiversity position, across all 3 indicators, as of the end of the most recent observation year (Table 1; Figure 4). Although operating over 50 sites, CSIRO's biodiversity position was overwhelmingly influenced by the very large Murchison site (Inyarrimanha Ilgari Bundara) (Appendix S6), where a small proportion of land is used for radioastronomy and the remainder has been historically used for extensive grazing. Although the average ecosystem condition across the Murchison site was estimated to be high (94%), relatively minor reductions in ecosystem condition over large areas added up to large total estimated change since CSIRO took control of the site (Figure 7; Appendix S6). The large reductions in the biodiversity position of CSIRO from 2009 to 2016 (Figure 4) are therefore primarily due to cattle grazing management on the Murchison site during that period. After 2016, stock were removed from the Murchison site, and the primary negative impacts there are now small areas of land clearing for new radioastronomy infrastructure.

Despite the dominance of the large Murchison site, the negative biodiversity position of CSIRO was consistent even when that site was excluded. The majority of CSIRO sites had a negative position when considered individually (Figure 5; Appendix S5). Only 11 of the 50 CSIRO sites were in a positive biodiversity position in terms of effective habitat area indicator (Figure 5; Appendix S2); only 3 sites contributed positively to threatened species habitat (Appendices S2 & S5); and no CSIRO sites effectively reduced the estimated extinction risk to indigenous species (Appendix S2). Although the magnitude of biodiversity impacts of different sites is most important, it may also be useful to consider the biodiversity position of each site relative to its area. When taking this relative view, several smaller CSIRO sites, such as Irymple and Merbein, were identified as having had relatively large negative impacts on biodiversity given their size (Figure 5).

The fine resolution (≈100 m) of the spatial data used as the basis for our organizational assessment enabled a more nuanced view for individual sites as to which areas in the site were responsible for the greatest estimated change and in which years across the site's history (Figure 6). This intrasite view could be used to help attribute estimated changes in the biodiversity indicators to specific past management actions. A finer view of the biodiversity indicators in a site could also be used to inform future management decisions, including identifying areas in which to focus habitat restoration or protection.

A benefit of a habitat‐based approach to organizational biodiversity assessment is that it provides a capacity to directly link past‐to‐present monitoring of biodiversity impact with present‐to‐future estimation of likely future biodiversity position under alternative scenarios of management. For CSIRO, we demonstrated this capability by assessing the potential benefits of implementing a natural regeneration management action across the large Murchison site, equivalent to removing all livestock grazing. We estimated that after 50 years, this action would return CSIRO's biodiversity position for Murchison to a positive biodiversity impact on effective habitat area (i.e., from −11,670 ha in 2024 to +392 ha in 2075) and bring CSIRO's total biodiversity position very close to neutral (Figure 7). Such forecasting capability could be used in sophisticated ways to help organizations set targets and understand the suite of actions that may be needed to achieve those targets (TNFD, 2023b).

### Limitations and future development

Although our general approach for estimating the direct impacts of an organization on biodiversity is conceptually sound (Figures 1 & 2), the rigor of its implementation depends on the accuracy of the data used. The accuracy of the time series of ecosystem condition data is particularly important for habitat‐based assessment approaches. We developed and applied fine‐resolution ecosystem condition data for Australia, which we validated using on‐ground data and which we believe represent the current best available data for this purpose. However, there are some locations where our estimated ecosystem condition departed from on‐the‐ground observations or a priori expectations. In addition, the ecosystem condition data we developed did not account for factors that were difficult to detect from space‐borne reflectance data but important determinants of ecosystem condition, including understory plant composition and structure, as well as non‐native invasive plant and animal species. Further research to understand the degree to which these factors are implicitly reflected in the estimated ecosystem condition values would be useful, as would improved methods for estimating ecosystem condition over time based on remotely sensed data. Whether the ecosystem condition data we applied are of sufficient accuracy for assessing impacts of other organizations depends upon the nature of their footprint, the tolerance of the information user, and the availability of more accurate alternative data sources.

Ongoing changes in biodiversity information can be incorporated into our assessment approach primarily by regenerating the full time series of spatial data based on the updated information. For example, changes in threatened species listings may result in new species being added to the data set on the potential distributions of threatened species, in which case the full historic time series of threatened species habitat should be generated with the additional data (Giljohann et al., 2025). Similarly, newly available biodiversity observations or species reclassifications can be utilized to update the models of community‐level biodiversity patterns, from which an updated extinction risk indicator can be derived. The analytical methods are well suited to regions with limited biodiversity data (Hoskins et al., 2020), so the same organizational assessment approach and indicators could be scaled to other regions or globally.

There are also likely to be benefits in extending habitat‐based assessment of the direct impacts of an organization beyond the 3 indicators that we have considered. A particular priority would be adding an indicator of ecosystem connectivity (Drielsma et al., 2022), given the importance of habitat configuration in influencing the ongoing persistence of species populations and ecosystem connectivity being considered by the TNFD as an important metric for organizational disclosure (TNFD, 2023d). We suggest that care needs to be taken in considering multiple biodiversity indicators together, given that an organization may be in a positive position for some indicators and not others. Combining multiple indicators into a single index (Zhu et al., 2024) is likely an undesirable solution, given that approach loses the inherent meaning of indicator values and may be disproportionately affected by a single component indicator.

Linking past‐to‐present monitoring of an organization's biodiversity position with future‐looking scenario analysis of proposed or implemented actions offers strong potential in helping organizations make decisions that benefit biodiversity (Nicholson et al., 2019). Our scenario assessment method relies on reasonably accurate assessment of the most recent ecosystem condition for an area and applies forecasting functions (Appendix S4) that generalize expected future outcomes based on relatively sparse available data on the observed impacts of different actions on biodiversity. There may be substantial variation in the actual response of ecosystem condition at a given location to the implementation of an action due to differences in the history of a site (e.g., weed invasion), local factors (e.g., soil attributes), the regional context (amount and configuration of surrounding habitat), or major climatic or disturbance events. Our forecasting functions would benefit from additional observed data in order to better represent these sources of regional variation. Future analyses could also explore and report the sensitivity of estimates of future actions to uncertainty in the initial ecosystem condition values and the response function parameters.

Finally, we considered only the direct impacts of CSIRO on biodiversity over the period of remotely sensed data (1987–2023) through impacts on ecosystem condition in terrestrial locations across Australia. We did not consider direct impacts on biodiversity for CSIRO sites prior to 1987 or the direct impacts at several small CSIRO sites located outside of Australia (France, Chile, the United States). We also did not include other direct impacts on biodiversity, such as on‐site pollution or impacts of off‐site activities (e.g., research surveys). Furthermore, we did not consider any indirect impacts of CSIRO on biodiversity, such as supply chains, waste, or effects of greenhouse gas emissions on biodiversity (Mokany et al., 2024). Future research could extend the approaches presented here to begin considering indirect impacts on biodiversity (Scope 3) (Figure 1) by incorporating information on the source location, amount and production intensity of input commodities, and their subcomponents, though this information is generally lacking globally (zu Ermgassen et al., 2022).

Broader application of our biodiversity assessment approach would need to consider a variety of practical social and governance issues. For example, although our approach attributes the historic direct biodiversity impacts to the organization responsible, ongoing application of this method would require careful management to avoid perverse or unintended outcomes, such as outsourcing or novation of negative impacts to shell companies. Robust management practice would be needed to account for changing ownership structures within and between companies to ensure accrued direct biodiversity impacts are appropriately attributed, including through company mergers, acquisitions, joint ventures, insolvency, and deregistration. Hence, rigorous biodiversity assessment approaches will need to be coupled with social and legal assurance, safeguards, and governance mechanisms to ensure they are used appropriately.

Organizational biodiversity assessment and reporting is a newly emerging and rapidly escalating need. To live up to its potential to help stem biodiversity declines (Leclère et al., 2020; SBTN, 2023; TNFD, 2023d), the data sources, analytical methods, and indicators applied need to be scientifically robust and fit for purpose. Comparative assessments with alternative assessment approaches may be required to determine the suitability and accuracy of different methods for different types of organizations in different regions. Scientifically rigorous methods are crucial for engendering trust in the biodiversity impacts reported by organizations.

## Supporting information



Supporting Information
