# Supplementary material for: A habitat‐based approach to reporting the direct impacts of an organization on biodiversity
Source: Conserv Biol. 2025 May 31;39(6):e70071. doi: 10.1111/cobi.70071 (PMC12658938; doi:10.1111/cobi.70071)
Supplement: Supplementary file 1 — Supporting Information [file COBI-39-e70071-s001.docx]

Supporting Information

**A habitat-based approach to reporting the direct impacts of an organisation on biodiversity**


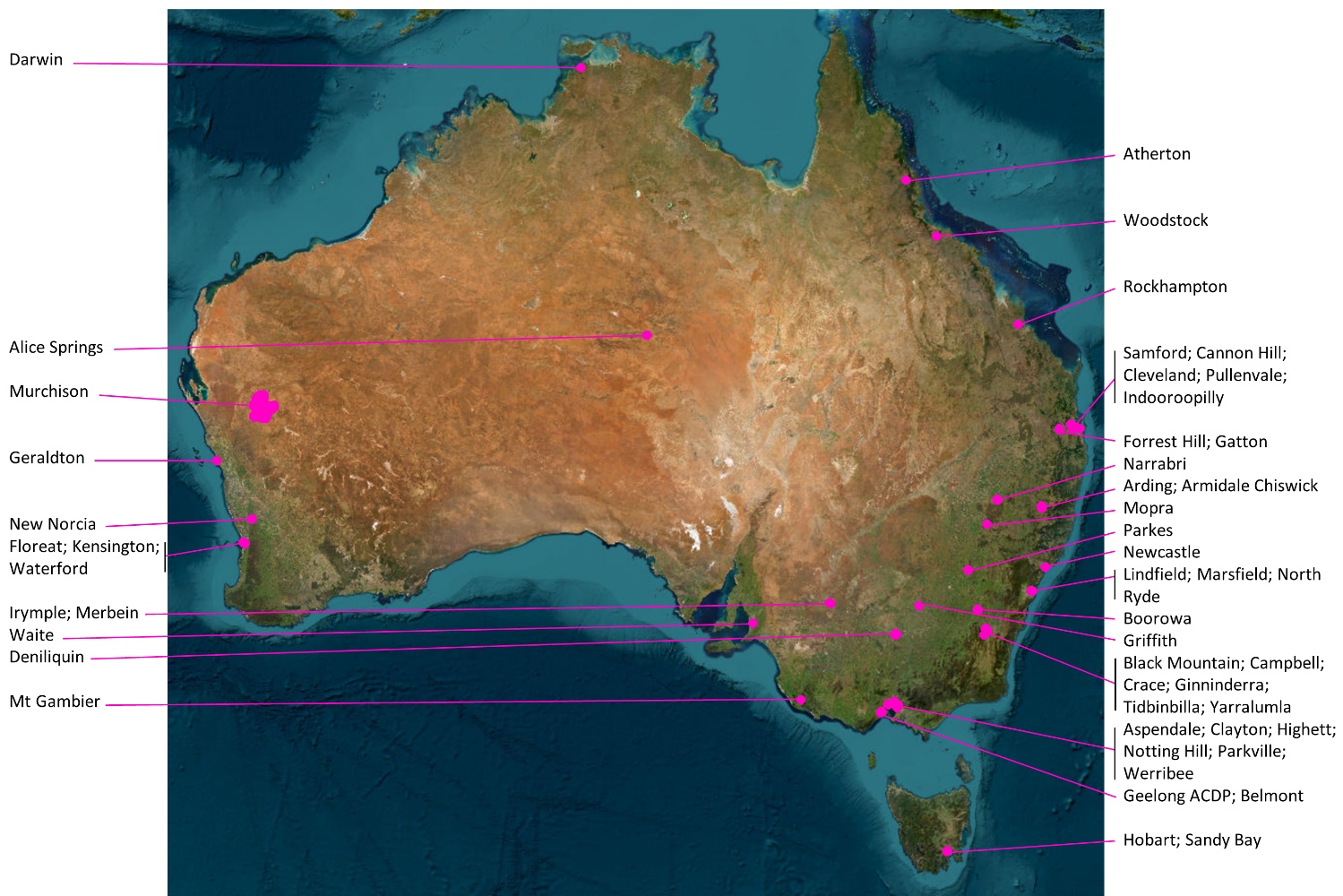


**Appendix S1.** Locations of the CSIRO sites used in the organisational biodiversity assessment.

**Appendix S2.** Estimated biodiversity position for each CSIRO site as of the most recent time with observation data (i.e. as of 1 January 2024). Start and end year indicate the years in which each site was acquired and divested, respectively.

| **Site** | **Start year** | **End year** | **Site area** (ha) | **Net change in species extinction risk**  (species) | **Net change in effective habitat area**  (ha) | **Net change in threatened species habitat**  (species.ha) |
| --- | --- | --- | --- | --- | --- | --- |
| Alice Springs | 1953 | 2009 | 37.9 | 0.00 | 0.0 | -0.5 |
| Arding | 1947 | 2020 | 321.9 | 0.00 | -1.7 | 0.0 |
| Armidale Chiswick | 1947 | NA | 1,494.7 | 0.00 | -20.5 | -285.5 |
| Aspendale | 1952 | NA | 2.0 | 0.00 | +0.0 | 0.0 |
| Atherton | 1971 | NA | 3.3 | 0.00 | -1.0 | -43.9 |
| Belmont | 1948 | 2020 | 6.2 | 0.00 | -1.2 | 0.0 |
| Black Mountain | 1927 | NA | 47.4 | +0.04 | -7.1 | -221.3 |
| Boorowa | 2019 | NA | 316.2 | 0.00 | -0.8 | 0.0 |
| Campbell | 1964 | 2016 | 3.9 | 0.00 | -0.8 | -41.1 |
| Cannon Hill | 1956 | 2008 | 6.0 | 0.00 | -0.6 | 0.0 |
| Clayton | 1961 | NA | 20.3 | 0.00 | +0.0 | -11.4 |
| Cleveland | 1974 | 2011 | 1.4 | 0.00 | -0.1 | 0.0 |
| Crace | 1953 | NA | 42.3 | 0.00 | -7.6 | -213.0 |
| Darwin | 1970 | NA | 21.4 | 0.00 | -0.8 | -14.9 |
| Deniliquin | 1944 | 1992 | 3,144.9 | 0.00 | -32.1 | -108.0 |
| Floreat | 1968 | NA | 11.3 | 0.00 | +0.1 | 0.0 |
| Forrest Hill | 2020 | NA | 74.9 | 0.00 | +0.2 | 0.0 |
| Gatton | 1930 | 2022 | 0.4 | 0.00 | 0.0 | 0.0 |
| Geelong ACDP | 1985 | NA | 35.7 | 0.00 | -6.9 | -72.8 |
| Geraldton | 2011 | NA | 0.2 | 0.00 | +0.0 | 0.0 |
| Ginninderra | 1958 | NA | 692.4 | +0.04 | -27.8 | -675.0 |
| Griffith | 1924 | 2016 | 10.6 | 0.00 | -1.0 | 0.0 |
| Highett | 1950 | 2020 | 9.2 | 0.00 | -1.1 | 0.0 |
| Hobart | 1981 | NA | 2.6 | 0.00 | -0.4 | 0.0 |
| Indooroopilly | 1962 | 2011 | 6.6 | 0.00 | -0.2 | -5.7 |
| Irymple | 1969 | NA | 15.3 | 0.00 | -8.0 | -94.5 |
| Kensington | 1999 | NA | 7.4 | 0.00 | -2.6 | -42.9 |
| Lindfield | 1968 | NA | 21.7 | 0.00 | +0.9 | -1.1 |
| Marsfield | 1957 | NA | 7.2 | 0.00 | -0.6 | -19.8 |
| Merbein | 1937 | 2012 | 34.2 | 0.00 | -14.7 | -200.6 |
| Mopra | 2005 | NA | 1.2 | 0.00 | +0.1 | +1.4 |
| Mt Gambier | 1975 | 2008 | 18.2 | 0.00 | -0.2 | +10.1 |
| Murchison | 2009 | NA | 451,703.9 | +0.81 | -11,669.7 | -18,704.8 |
| Narrabri | 1963 | NA | 1,241.3 | 0.00 | +21.9 | +193.9 |
| New Norcia | 2018 | NA | 3.7 | 0.00 | -0.2 | 0.0 |
| Newcastle | 1999 | NA | 5.1 | 0.00 | -0.1 | 0.0 |
| North Ryde | 1996 | NA | 1.3 | 0.00 | +0.1 | 0.0 |
| Notting Hill | 1991 | NA | 0.1 | 0.00 | 0.0 | 0.0 |
| Parkes | 1961 | NA | 160.8 | 0.00 | -6.7 | -154.5 |
| Parkville | 1950 | 2022 | 0.5 | 0.00 | -0.0 | 0.0 |
| Pullenvale | 1991 | NA | 21.1 | 0.00 | -5.3 | -166.9 |
| Rockhampton | 1973 | 2011 | 32.8 | 0.00 | -2.0 | -35.7 |
| Samford | 1959 | 2002 | 286.4 | 0.00 | -5.4 | -169.4 |
| Sandy Bay | 1999 | NA | 1.1 | 0.00 | -0.1 | -1.1 |
| Tidbinbilla | 1965 | NA | 53.1 | 0.00 | -0.8 | -13.0 |
| Waite | 1970 | NA | 3.0 | 0.00 | -0.8 | -11.7 |
| Waterford | 2010 | NA | 2.9 | 0.00 | +0.1 | 0.0 |
| Werribee | 2014 | NA | 12.5 | 0.00 | +0.1 | 0.0 |
| Woodstock | 1962 | NA | 632.9 | +0.20 | -139.6 | -1,179.6 |
| Yarralumla | 1975 | 2022 | 11.7 | 0.00 | -0.4 | -23.7 |

**Appendix S3 – Deriving spatial estimates of ecosystem condition from remotely sensed data**

Spatial data on ecosystem condition across Australia were generated by combining two approaches that are applied in areas either naturally expected to have tree cover, or in areas with very sparse or no natural tree cover.

For areas in Australia expected to naturally have tree cover, ecosystem condition was derived using a rule-based approach with the key remotely-sensed input being the National Forest and Scattered Woody annual layers (DCCEEW, 2021a), developed and used for Australia’s national carbon accounting system. The time series of tree cover in each 25 m grid cell was combined with information on natural disturbance to tree cover from the MODIS burned area time series (2001-2023) (Giglio *et al.*, 2015). Other spatial information was also used to adjust ecosystem condition in areas that had persistent tree cover but where axillary information on land use indicated likely low ecosystem condition, including areas identified as exotic softwood plantations (ABARES, 2016) and urban areas (Geoscience Australia, 2006). The data for each year were then resampled to 0.001 degree grid resolution (≈ 100 m).

To quantify ecosystem condition for areas expected to naturally have very little or no tree cover, we used the ‘Compere’ approach (Donohue *et al.*, 2022). The remotely sensed data applied were annual layers (1987−2023) of Normalised Difference Vegetation Index (NDVI) for Australia (Geoscience Australia, 2021), as percentages (where NDVI = 1 equates to 100 %). A key aspect of Compere is the comparison of a vegetation attribute in a target location (e.g. grid cell) with the same attribute in all other biophysically equivalent locations. To identify biophysically equivalent locations, we classified all locations (grid cells) across Australia using a manual interval classification (250 classes) based on four environmental layers: (i) soil sand content (0−200 cm depth) (Viscarra Rossel *et al.*, 2015), (ii) potassium radiometric concentration (Wilford and Kroll, 2019), (iii) lakes and swamps derived from the Australian geofabric (BOM, 2012), and (iv) topographic wetness derived as the product of topographic wetness index (Gallant and Austin, 2012) multi-resolution valley bottom flatness (Gallant and Austin, 2015) and contributing area (Gallant and Austin, 2015).

To account for regional difference in recent rainfall history, we implemented the Compere analysis separately for spatial blocks of size 25 x 25 km across Australia. Within each spatial block, for each year separately, the NDVI in each grid cell was compared to the values for all other grid cells in the same environmental category (from the environmental classification) in that block in that year. The 85^th^ percentile of NDVI values in each environmental category in each block in each year were used as the relative benchmark (Donohue *et al.*, 2022), which inherently assumes that at least 15 % of grid cells within every environment category within every 25 km block are in or close to reference condition. This may be justified, given our application of Compere was only for areas with naturally low or no natural tree cover, which in Australia are very commonly areas of native vegetation. Any locations in naturally treeless environments that are dominated by alien invasive plant species are likely to have overestimated ecosystem condition values. Using the NDVI value (*V_i,tw_*) of the target grid cell *i* in year *w* (*t_w_)*, and the relative benchmark of NDVI for that environmental category in that spatial block in year *w* (*V_B,tw_*), the inferred ecosystem condition for the target grid cell (*c_i,tw_*) was derived as:

$$c_{i,tw}=\frac{1-e^{-3\left( \frac{V_{i,tw}}{V_{B,tw}} \right)}}{1-e^{-3}}$$

The negative exponential function applied above transforms the fraction of the target grid cell relative to the benchmark (*V_i,t_* / *V_B,t_*) into a non-linear response, where inferred ecosystem condition decreases more when the NDVI of the target cell is lower.

The two ecosystem condition datasets, based on whether tree cover was expected or not, were combined into a single spatial layer of ecosystem condition for Australia for each year (Figure S2). This was based on the environments in which we expect to have natural tree cover, for which we used a layer of potential plant growth index (Xu and Hutchinson, 2010) and mapped pre-European areas of non-tree vegetation types (DCCEEW, 2021b).


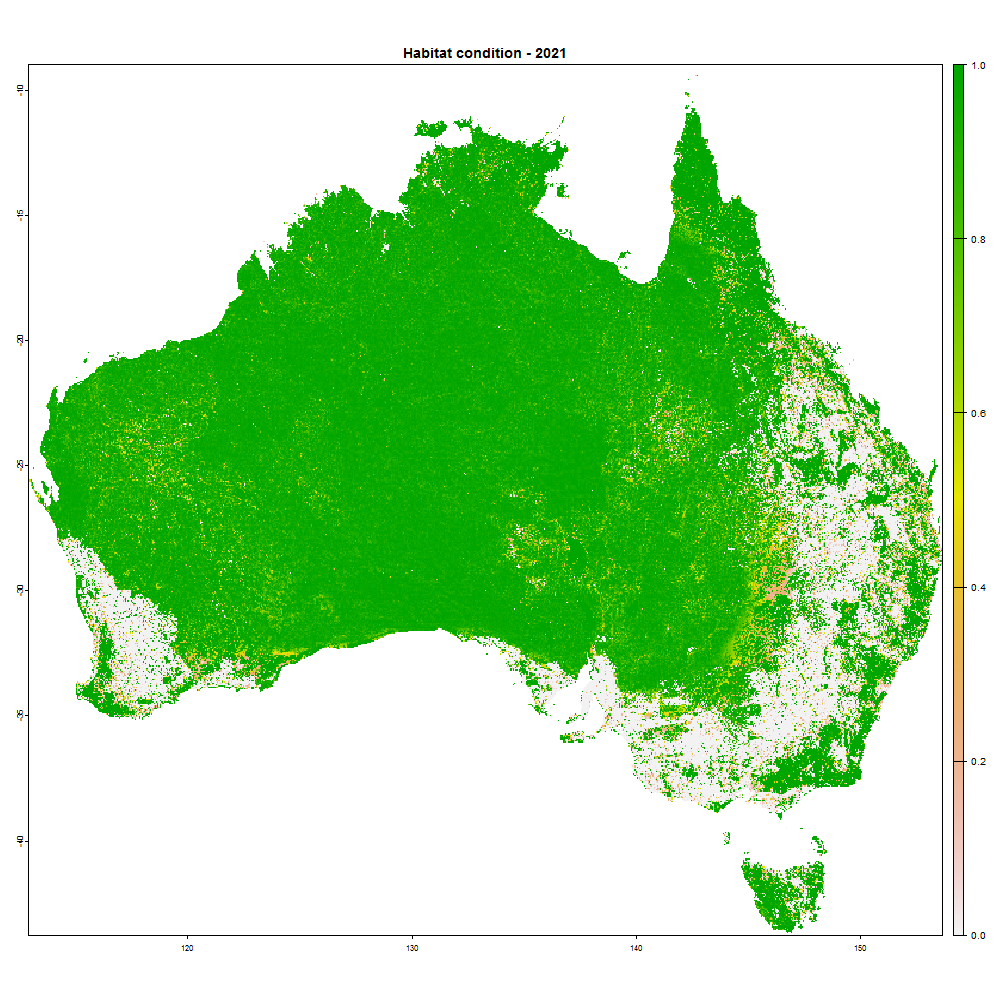
**Figure S2.** The mapped ecosystem condition data for Australia, shown for the most recent observation year (2023). The scale shown is on a 0−1 range, where a value of ‘1’ indicated reference condition, and a value of ‘0’ indicates fully degraded condition.

To validate the annual ecosystem condition spatial layers produced, we used several continent-wide datasets from which an estimate of ecosystem condition can be inferred. The primary source of data for validation were plant community survey plot data from across Australia (Mokany *et al.*, 2022), from which the proportion of species present that were native to Australia was used as the proxy condition index (n = 183,382 survey locations). Given most plant community surveys are undertaken in relatively intact vegetation (though does include some intermediate values; Figure S3), these data were supplemented with data for locations that were assumed to be fully degraded (inferred ecosystem condition = 0). Fully degraded locations were inferred from spatial datasets of motorways, carparks and buildings (OpenStreetMap, 2021), vineyards (OpenStreetMap, 2021), tree crops (Australian Tree Crop Map, 2020) and cultivated agricultural fields (Owers *et al.*, 2021). Softwood plantations (ABARES, 2016) were also considered, but assumed to have an ecosystem condition value of 0.3 (i.e. 30 %). From these spatial datasets a spatially stratified random selection of points (n = 70,000) was selected across Australia’s bioregions (Thackway and Cresswell, 1995) which were then combined with the plant community survey data. To assess the predictive accuracy of the ecosystem condition layers, we extracted the predicted condition values for the matching location and year of each observation in the validation data. The annual estimated ecosystem condition layers performed moderately well in predicting observed ecosystem condition (R^2^ = 0.511; mean absolute error = 0.173; root mean square error = 0.292).


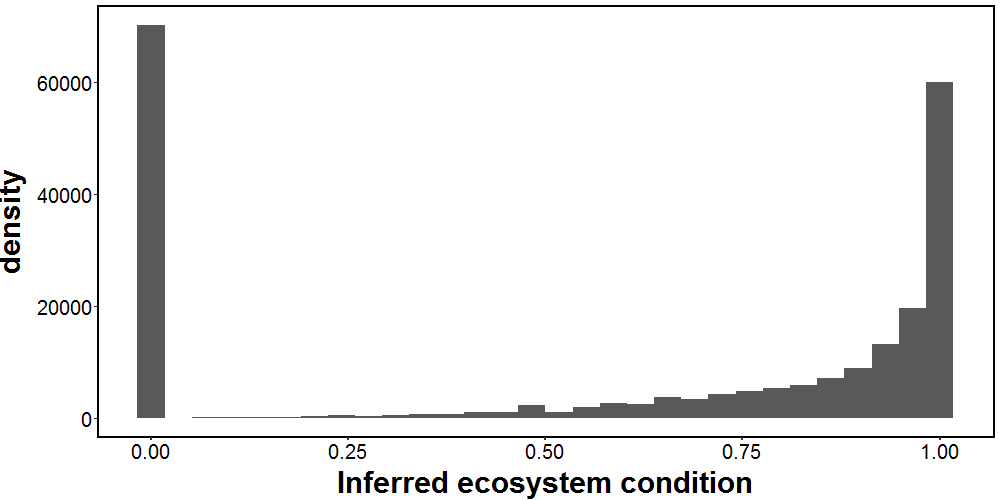


**Figure S3.** The distribution of inferred ecosystem condition values for locations used to validate the mapped ecosystem condition data for Australia. The ecosystem condition data are shown on a 0−1 range, where a value of ‘1’ indicated reference condition, and a value of ‘0’ indicates fully degraded condition.

**Appendix S4 – Forecasting future ecosystem condition under alternative ecosystem management actions**

Future estimates of ecosystem condition take the most recent observed estimate available for each grid cell in the analysis area as the starting point. Ecosystem condition (expressed as a percentage) for a grid cell *i* in *t_w_* years after an action was implemented (*c_i,tw_*) is predicted using a four-parameter logistic model:

$$c_{i,tw}=d+\frac{c_{i,t0}-d}{1+\left( \frac{t_{w}}{h} \right)^{b}}$$

where *c_i,t0_* is the initial ecosystem condition for location *i*, and parameters *b*, *h*, and *d* are dependent on the action implemented (Table S2). Importantly, the maximum achievable ecosystem condition (*d*) for a grid cell depends on the initial ecosystem condition:

$$d=g+\left( v-g \right)\left( 1-e^{-kc_{i,t0}} \right)$$

which accounts for the difficulty of improving ecosystem condition to high levels in highly degraded locations. Parameters *g*, *v* and *k* are again dependent on the action implemented (Table S2).

The types of actions considered in forecasting future ecosystem condition include:

- **Environmental planting**: equivalent to a diverse, mixed native species restoration planting and associated soil remediation.
- **Plantation forestry (native)**: a monoculture even-aged forestry plantation of a native tree species;
- **Plantation forestry (exotic)**: a monoculture even-aged forestry plantation of a non-native tree species;
- **Natural regeneration**: the removal of any anthropogenically-derived disturbance (e.g. livestock grazing, non-natural fire regime, weed or feral species) that enables the ecosystem to recover naturally;
- **Land clearing**: the removal of all native vegetation from an area.

**Table S2**. Parameters applied in the ecosystem condition forecast method for each action type.

| **Action** | ***b***  (slope) | ***k***  (scalar) | ***h***  (number of years to achieve half the potential change) | ***v***  (maximum potential final habitat condition) | ***g***  (minimum potential final habitat condition) |
| --- | --- | --- | --- | --- | --- |
| Environmental planting | 2 | 0.1 | 25 | 80 | 60 |
| Plantation forestry (native) | 2 | 0.1 | 25 | 60 | 50 |
| Plantation forestry (exotic) | 2 | 0.1 | 25 | 30 | 30 |
| Natural regeneration | 1.1 | 0.21 | 24 | 98 | 21 |

When depicted graphically (Fig. S4), it is evident that the ecosystem condition forecast functions are flexible in allowing for both different rates of change, and potential maximum values, depending on the initial ecosystem condition value.


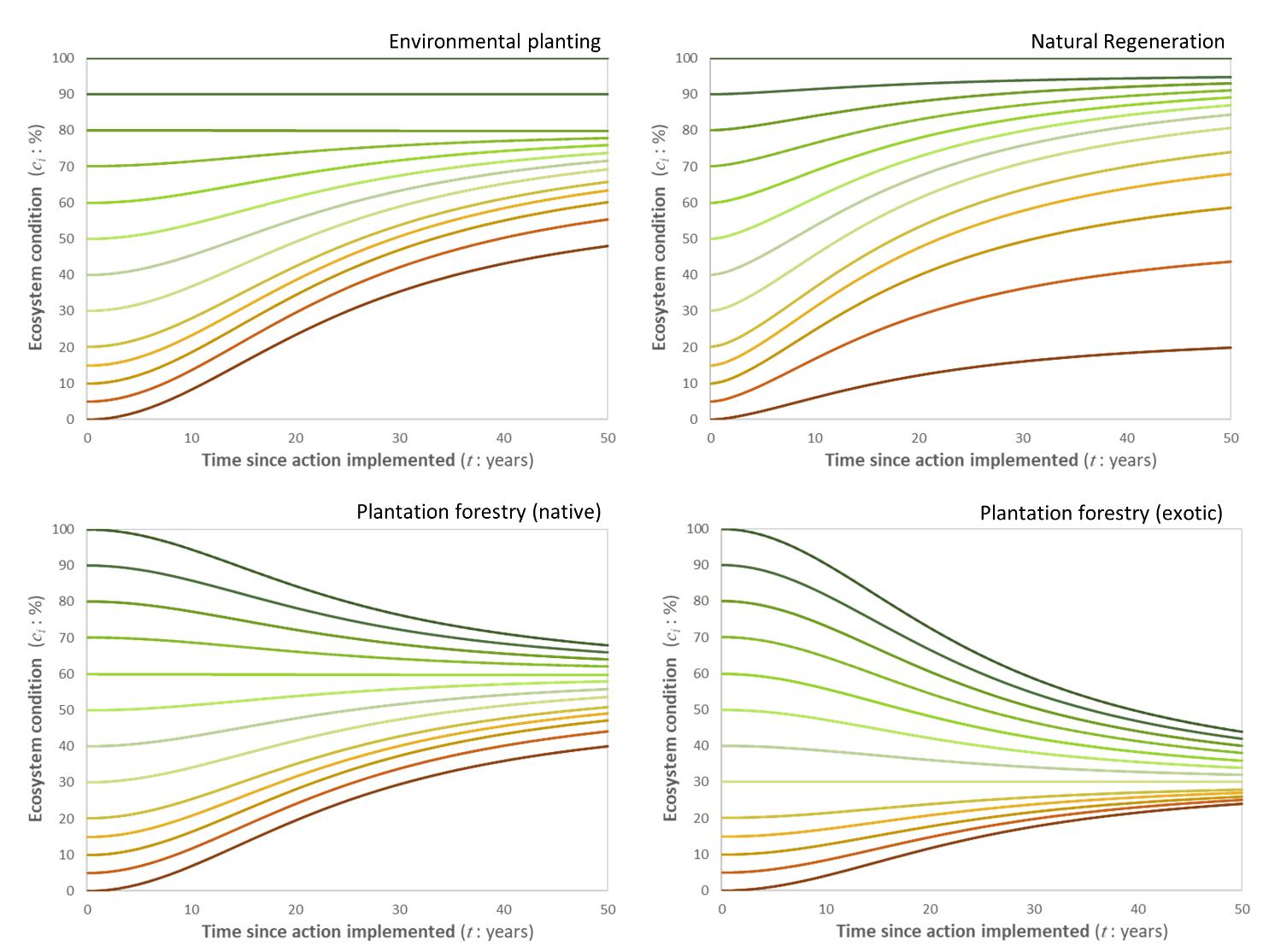


**Figure S4.** Graphical depiction of the forecasted changes in ecosystem condition for four different ecosystem management actions, given differences in initial ecosystem condition (at *t*_0_) shown with different line colour, based on the model and parameters applied (Table S2).

The parameters for maximum potential final ecosystem condition (*v*) and minimum potential final ecosystem condition (*g*) for the environmental planting, plantation forestry (native) and plantation forestry (exotic) action types were selected based on a review of observed biodiversity outcomes from different tree planting actions in agricultural landscapes (Prober *et al.*, 2024). This review considered published studies that measured ecological community composition under all of three contrasting land uses within a landscape: (i) a natural (reference) system (e.g. forest or woodland); (ii) a non-woody agricultural system (crop or pasture), and; (iii) a former agricultural system that has had woody plants established on it (environmental planting or plantation forestry). From each study, data on the difference in species composition between each land use was obtained, primarily by digitizing ordination plots, but also through directly reported compositional similarities. From these observed compositional differences, we calculated the relative similarity of the woody planting to the natural reference system (*s_rel_*) as:

$s_{rel}=1-\frac{d_{rp}}{d_{ra}}$

where *d_rp_* is the compositional difference between the natural reference system (*r*) and the woody planting (*p*), and *d_ra_* is the compositional difference between the natural reference system (*r*) and the agricultural system (*a*).

We restricted the data to studies from Australia and for woody plantings that were ≥ 10 years old. The average relative similarity (*s_rel_*) of environmental plantings to the natural reference system was 0.36 (n = 13, SD = 0.25). The average relative similarity (*s_rel_*) of native plantation forestry to the natural reference system was 0.27 (n = 3, SD = 0.27). There were insufficient data to obtain the average relative similarity for exotic plantation forestry ≥ 10 years old.

To apply the average relative similarities obtained from the literature review in parameterising the models of changes in ecosystem condition over time following implementation of ecosystem management actions (Table S2), we set the maximum potential final ecosystem condition (*v*) and minimum potential final habitat condition (*g*) values such that the estimated ecosystem condition at *t* = 25 years (*c_i,t_*_25_) was as predicted by the given *S_rel_* value and the ecosystem condition at *t* = 0 (*c_i,t_*_0_):

$$c_{i,t25}=c_{i,t0}+\left( s_{rel}\left( 100-c_{i,t0} \right) \right)$$

For the plantation forestry (exotic) management action, the ecosystem condition model parameters (Table S2) were estimated based on expert judgement, in the context of the parameters derived for environmental plantings and plantation forestry (native).

To parameterise the function to predict future ecosystem condition under the natural regeneration management action, we searched extensively (Web of Science) for published studies that: (i) occurred in Australia on land with a prior history of agricultural grazing and/or complete clearing; (ii) measured biodiversity composition at a grazed/cleared site, an ungrazed natural reference site, and at naturally regenerating site/s with known time since cessation of grazing/clearing, and; (iii) consider natural regeneration without the addition of other management actions, such as planting. The grazed site/s are used as the baseline initial ecosystem condition, and the ungrazed natural reference site/s used as the reference point for similarity comparisons. For each of the 15 studies that presented suitable community composition data, we used the compositional similarity to the natural reference site/s as the measure of ecosystem condition for both grazed site/s (assumed t=0 years since regeneration), and the naturally regenerating site/s (capturing also the time since regeneration commenced). These data were used to fit the parameters for the logistic model for natural regeneration (Table S2), with the resulting parameters when applied in the function having a root mean square error of 11.6 % for the training data.


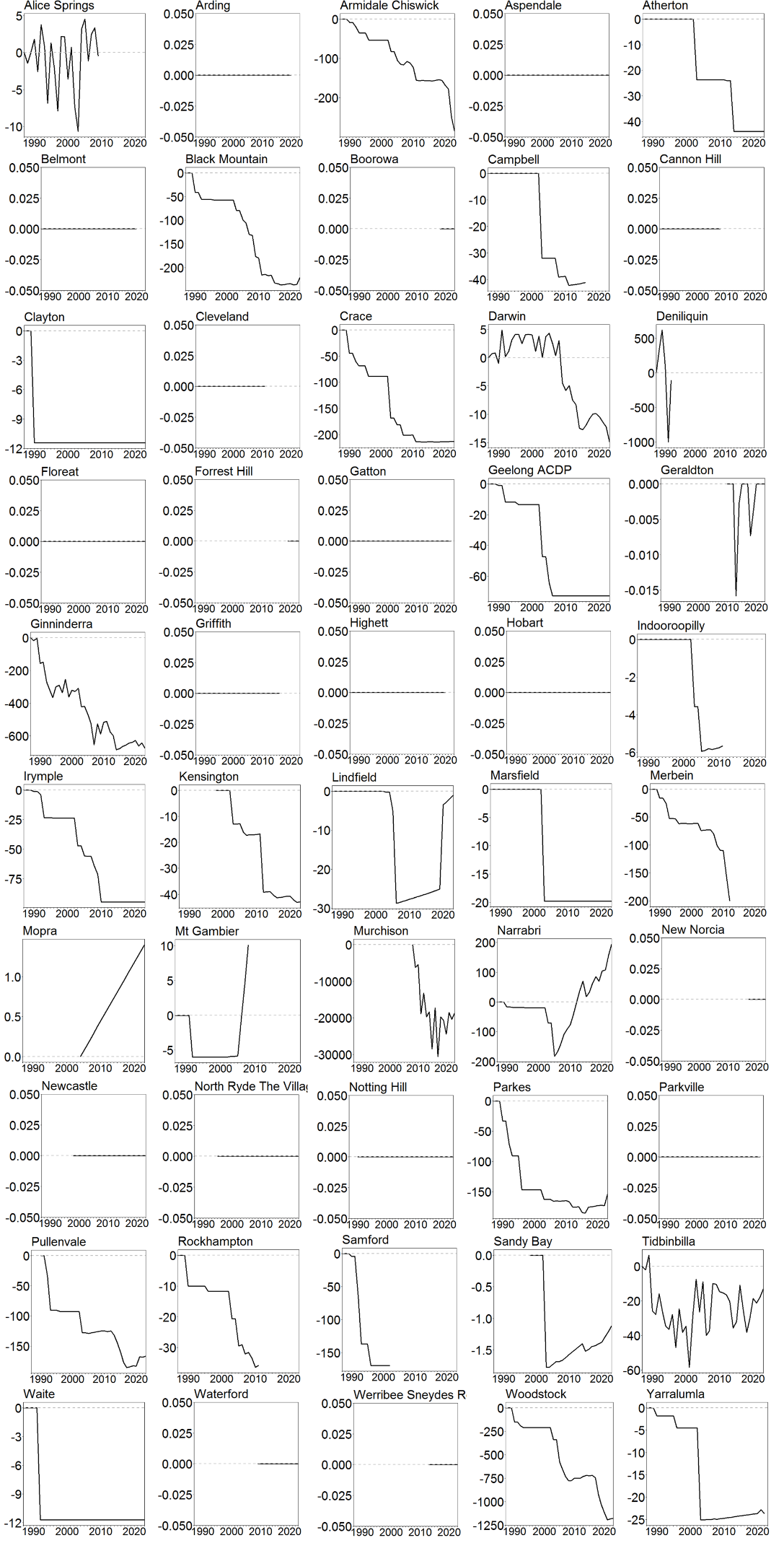


**Appendix S5.** Threatened species habitat time series for each of the 50 CSIRO sites.


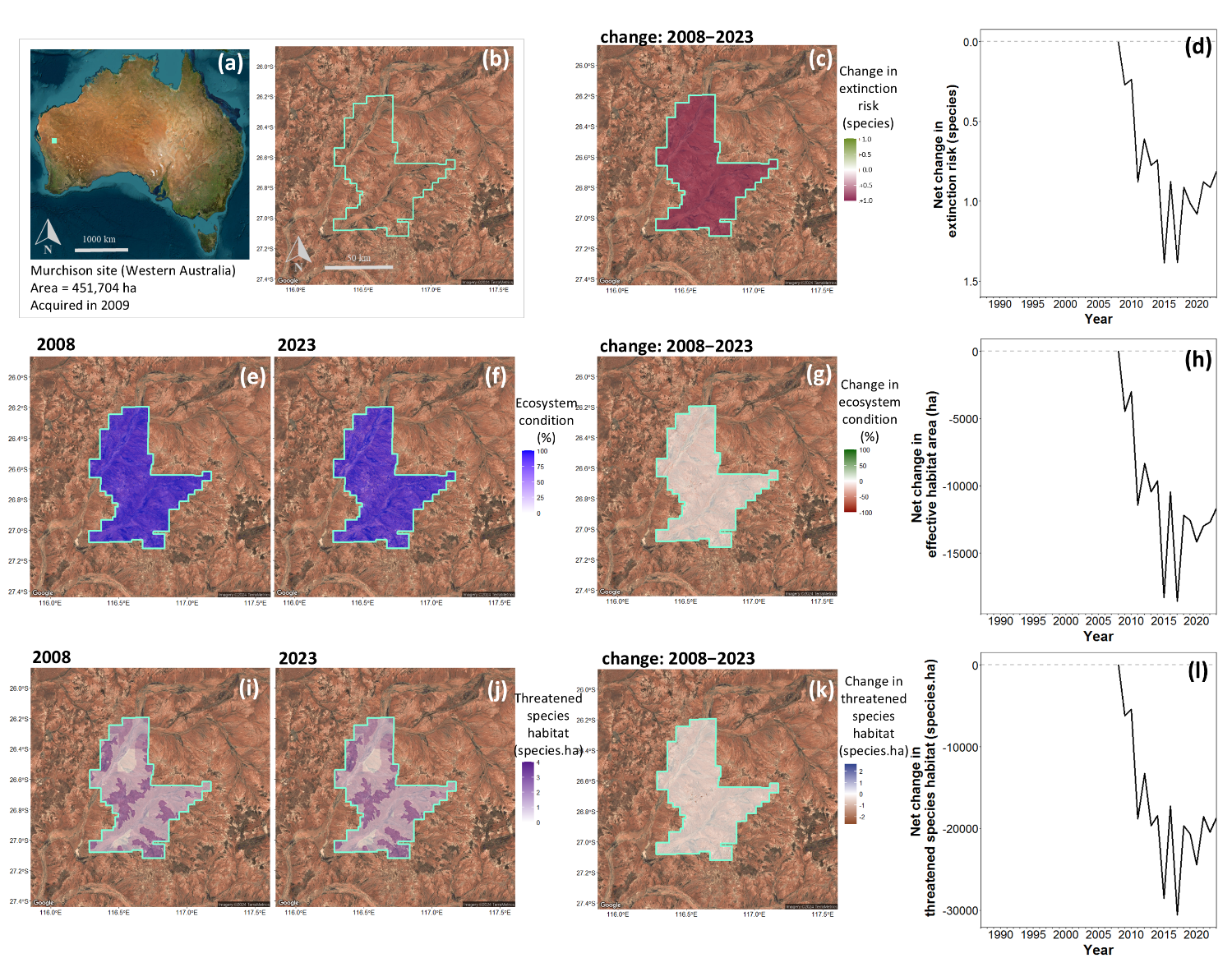


**Appendix S6.** Site-level biodiversity assessment for CSIRO’s Murchison site in Western Australia, including contextual information (**a**., **b**.). Outcomes are shown for three biodiversity indicators: species extinction risk (**c**. **d**.); ecosystem condition (and effective habitat area) (**e**.−**h**.), and; threatened species habitat (**i**.−**l**.). Maps show raster data for the baseline year for this site (2008: **e**., **i**.), the most recent available year (2023: **f**., **j**.) and the change over the time series (**c**., **g**., **k**.). Change in the site-level biodiversity position for the three indicators over time is also shown (**d**., **h**., **l**.). Background imagery - Map data ©2024 Google.

**Appendix S7.** Definition of terms used in the equations.

| **Equation term** | **Definition** | **Units** |
| --- | --- | --- |
| *a_i_* | The area of grid cell *i* | hectares (ha) |
| *c_i,t_* | The estimated ecosystem condition for grid cell *i* in year *t*, expressed in a 0−1 range |  |
| *c_i,tw,count_* | The counterfactual ecosystem condition value for grid cell *i* in year *t_w_* |  |
| *E_n_* | The expected number of originally occurring native species across the region that are at risk of extinction | species |
| *E_p_* | The expected proportion of originally occurring native species across the region that are at risk of extinction |  |
| *E_n,count_* | The estimated number of species at risk of extinction across the entire region based on the counterfactual spatial patterns in ecosystem condition, where the organisation has not altered ecosystem condition | species |
| *E_n,obs_* | The estimated number of species at risk of extinction across the entire region based on the observed spatial patterns in ecosystem condition | species |
| *E_n,org_* | The number of species at risk of extinction due to the activities of the organisation (*org*) | species |
| γ | The estimated number of originally occurring (native) species in the analysis region | species |
| *H_k,t_* | The effective habitat area (area weighted condition) within polygon *k* in year *t* | hectares (ha) |
| ∆*H_k,tw_* | The net change in effective habitat area for polygon *k* in year *w* (*t_w_*) | hectares (ha) |
| ∆*H_tw_* | The net cumulative change in effective habitat area in year *w* (*t_w_*) across all polygons in the organisation’s footprint | hectares (ha) |
| *m_i_* | The number of species expected to occur in grid cell *i* (i.e. species richness) | species |
| *p_i_* | The proportion of native species historically occurring in cell *i* (pre-intensification) that are likely to persist within remaining habitat anywhere in their range given spatial patterns in ecosystem condition |  |
| *P* | The proportion of species expected to persist across a region given spatial patterns in ecosystem condition |  |
| *q_i_* | The number of threatened species for which grid cell *i* could potentially form part of their habitat | species |
| *s_ij_* | The predicted compositional similarity between the grid cell *i* and grid cell *j* |  |
| Σ*s_ij_* | The amount of similar habitat across the region; i.e. the summed similarity between grid cell *i* and all other grid cells *j* in the region |  |
| *t*_0_ | The year prior to the organisation taking control of a polygon (i.e. the baseline year) |  |
| *t_e_* | The year that the organisation relinquishes control of a polygon |  |
| *t_w_* | Year *w*; the year that an indicator is derived for |  |
| *T_i,t_* | The capacity of habitat to support threatened species in grid cell *i* in year *t* | species hectares (species.ha) |
| *T_k,t_* | The estimated capacity of habitat to support threatened species across all grid cells within a polygon *k* in any year *t* | species hectares (species.ha) |
| ∆*T_k,tw_* | The net change in threatened species habitat for polygon *k* from the baseline year (*t*_0_) to year *t_w_* | species hectares (species.ha) |
| ∆*T_tw_* | The net cumulative change in threatened species habitat across all polygons in the organisation’s spatial footprint, in year *t_w_* | species hectares (species.ha) |
| *v_i_* | A weighting of the relative importance of grid cell *i*, based on its species richness (*m_i_*) and the amount of similar habitat across the region (Σ*s_ij_*) |  |
| *z* | The exponent of the species-area relationship (power model) |  |

**References**

ABARES, 2016. Australia’s plantations 2016 dataset. Australian Bureau of Agricultural and Resource Economics and Sciences – (ABARES),, <https://www.agriculture.gov.au/abares/forestsaustralia/forest-data-maps-and-tools/spatial-data/australias-plantations>

Australian Tree Crop Map, 2020. The Australian Tree Crop Map Dashboard. Australian Tree Crop Map,

BOM, 2012. Australian Hydrological Geospatial Fabric (Geofabric). Version 2.1. Commonwealth of Australia (Bureau of Meteorology).

DCCEEW, 2021a. National Forest and Scattered Woody Vegetation Data (Version 5.0 - 2020 Release). Australian Government Department of Climate Change, Energy, the Environment and Water, <https://data.gov.au/dataset/ds-dga-69d09a6c-df77-439f-8bc7-87822cd520fd>

DCCEEW, 2021b. National Vegetation Information System (NVIS) Version 6.0—Australia- Estimated Pre-1750 Vegetation [Grid]. Australian Government Department of Climate Change, Energy, the Environment and Water, <https://data.gov.au/dataset/ds-environment-5556c707-a7f8-4d36-9262-f79a9f75ca92/details?q=National%20Vegetation%20Information%20System%20(NVIS)%20Version%206.0%E2%80%94Australia-%20Estimated%20Pre-1750%20Vegetation>

Donohue, R.J., Mokany, K., McVicar, T.R., O'Grady, A.P., 2022. Identifying management-driven dynamics in vegetation cover: Applying the Compere framework to Cooper Creek, Australia. Ecosphere 13, e4006.

Gallant, J., Austin, J.M., 2012. Topographic Wetness Index derived from 1" SRTM DEM-H. v2. CSIRO Data Collection. <https://doi.org/10.4225/08/57590B59A4A08>

Gallant, J.C., Austin, J.M., 2015. Derivation of terrain covariates for digital soil mapping in Australia. Soil Research 53, 895-906.

Geoscience Australia, 2006. GEODATA TOPO 250K Series 3 Topographic Data. Geoscience Australia,, <http://www.ga.gov.au:88/newintranet/meta/ANZCW0703005458.html>

Geoscience Australia, 2021. DEA Geometric Median and Median Absolute Deviation (Landsat). Geoscience Australia,, <https://cmi.ga.gov.au/data-products/dea/645/dea-geometric-median-and-median-absolute-deviation-landsat>

Giglio, L., Justice, C., Boschetti, L., Roy, D., 2015. MCD64A1 MODIS/Terra+Aqua Burned Area Monthly L3 Global 500m SIN Grid V006. NASA EOSDIS Land Processes DAAC,, <https://doi.org/10.5067/MODIS/MCD64A1.006>

Mokany, K., McCarthy, J., Falster, D., Gallagher, R., Harwood, T.D., Kooyman, R., Westoby, M., 2022. Harmonised Australian Vegetation Plot dataset (HAVPlot). CSIRO, <https://doi.org/10.25919/5cex-4s70>

OpenStreetMap, 2021. OpenStreetMap. OpenStreetMap

Owers, C.J., Lucas, R.M., Clewley, D., Planque, C., Punalekar, S., Tissott, B., Chua, S.M.T., Bunting, P., Mueller, N., Metternicht, G., 2021. Living Earth: Implementing national standardised land cover classification systems for Earth Observation in support of sustainable development. Big Earth Data 5, 368-390.

Prober, S., Liedloff, A.C., England, J.R., Mokany, K., Ogilvy, S., Richards, A.E., 2024. Accounting for the biodiversity benefits of woody plantings in agricultural landscapes: a global meta-analysis. Agriculture, Ecosystems & Environment in review.

Thackway, R., Cresswell, I.D., 1995. An Interim Biogeographic Regionalisation for Australia: A framework for establishing the national system of reserves. Australian Nature Conservation Agency,, Canberra, Australia

Viscarra Rossel, R.A., Chen, C., Grundy, M.J., Searle, R., Clifford, D., Campbell, P.H., 2015. The Australian three-dimensional soil grid: Australia’s contribution to the GlobalSoilMap project. Soil Research 53, 845-864.

Wilford, J.R., Kroll, A., 2019. Complete Radiometric Grid of Australia (Radmap) v4 2019 with modelled infill. Geoscience Australia,, <https://dev.ecat.ga.gov.au/geonetwork/srv/api/records/144413>

Xu, T., Hutchinson, M., 2010. ANUClim Version 6.1 User Guide. Fenner School of Environment and Society - The Australian National University,, Canberra
